# Solid stress compression enhances breast cancer cell migration through the upregulation of Interleukin-6

**DOI:** 10.3389/fcell.2025.1541953

**Published:** 2025-04-30

**Authors:** Farouq Azizan, Ryna Shireen Sheriff, Corinna Jie Hui Goh, Keng Hwee Chiam, Cheng-Gee Koh

**Affiliations:** ^1^ School of Biological Sciences, Nanyang Technological University, Singapore, Singapore; ^2^ Bioinformatics Institute, Agency for Science, Technology and Research (A*Star), Biopolis, Singapore, Singapore

**Keywords:** solid stress compression, cell migration, Interleukin-6, mechanosensitive genes, transcriptome

## Abstract

Apart from biochemical signals, tumour cells respond to biophysical and mechanical cues from their environment. The mechanical forces from the tumour microenvironment could be in the form of shear stress, tension, or solid stress compression. In this study, we explore the effects of solid stress compression on tumour cells. Solid stress compression, a prevalent biomechanical stimulus accumulated during tumour growth, has been shown to enhance invasive and metastatic phenotypes in cancer cells. However, the underlying molecular mechanism that elicits this aggressive metastatic phenotype, especially in breast cancer, is not extensively studied. Using an established 2D *in vitro* setup to apply incremental solid stress compression, we found that migratory and invasive capacities of aggressive breast cancer cells were enhanced in a biphasic manner. We also found that the transcript and protein levels of Interleukin-6 (IL-6) and SNAI1 were upregulated in response to solid stress. The resultant increased secretion of IL-6 could in turn lead to autocrine activation of downstream signalling pathways and impact on cancer cell migration and invasion.

## Introduction

Solid stress is the amount of physical force exerted per unit area which, in the context of a tumour mass, is the accumulation of physical forces exerted on cells of tumour mass as a result of uncontrolled growth and expansion in size within the finite space of the body (Kalli and Stylianopoulos, 2018). Essentially, as a tumour mass expands rapidly within the body, it pushes on the surrounding tissues which then returns an equal and opposite force back onto the tumour mass known as solid stress compression (Tse et al., 2012; Kalli and Stylianopoulos, 2018). This is particularly felt by the cells at the peripheral layer of the tumour mass, which are most likely to undergo invasion and metastasis (Tse et al., 2012). Earlier studies show that excessive solid stress pressures inhibited tumour growth and induced apoptosis (Cheng et al., 2009). However, recent studies have reported that under lower and more pathophysiological relevant solid stress pressures, cancer cells exhibit enhanced metastatic phenotypes ranging from increased cell migration, invasion and induction of epithelial to mesenchymal transition (EMT) (Demou, 2010; Tse et al., 2012; Fernandez-Sanchez et al., 2015; Chen et al., 2017; Kalli et al., 2019; Luo et al., 2022). More specifically, in breast cancer cells, solid stress compression at 773.0 Pa (5.8 mmHg), the estimated breast tumour microenvironment pressure, enhanced cell migration in aggressive breast cancer cell lines through the formation of “leader cells” at the leading edge or cell border (Tse et al., 2012). Furthermore, triple negative breast cancer cells under compression at 200.0 Pa, 400.0 Pa and 600.0 Pa, exhibited more invasive phenotypes through enhanced matrix degradation as a consequence of Piezo1 activation (Luo et al., 2022). In human clear cell renal cell carcinomas (ccRCC), solid stress of 534.0 Pa enhanced cell migration and when in combination with Interleukin-6 (IL-6) resulted in the induction of EMT through the Akt/GSK-3β/β-catenin signalling pathway (Chen et al., 2017). Compressed pancreatic cancer cells demonstrated greater cancer migratory potential under 534.0 Pa of solid stress through 1) the activation of Akt/CREB1/GDF15 pathway, 2) the activation of both the p38 MAPK/HSP27 and JNK/c-Jun signalling pathways and 3) the upregulation of key actin cytoskeleton modulators including CDC42 and RAC1 (Chen et al., 2017; Kalli et al., 2022). In an ambitious study where magnetically generated compression of 1200.0 Pa was applied *in vivo on* healthy colon epithelial cells of mice using magnetic beads, tumorigenesis occurred through the activation of Ret kinase and subsequent Akt/GSK-3β/β-catenin signalling pathway resulting in the upregulation of genes such as c-MYC (Fernandez-Sanchez et al., 2015). These studies accentuate the malignant role of solid stress compression in exacerbating cancer progression through the activation of different signalling pathways and gene upregulation.

In this study, we decipher how breast cancer cells react to increasing solid stress pressures because a rapidly expanding tumour mass would experience incremental levels of solid stress pressures throughout the course of tumour progression (Kalli and Stylianopoulos, 2018). We investigated the effects of incremental solid stress compression, 386.5 Pa, 773.0 Pa and 1546.0 Pa, on the global transcriptomic and metastatic phenotype alterations in an aggressive breast cancer cell line, MDA-MB-231. We aim to identify mechanosensitive genes and signalling pathways implicated in MDA-MD-231 cellular responses and behaviour under compressive stress.

## Results

### Incremental solid stress pressures activate Akt signalling pathway in breast cancer cells

**FIGURE 1:**
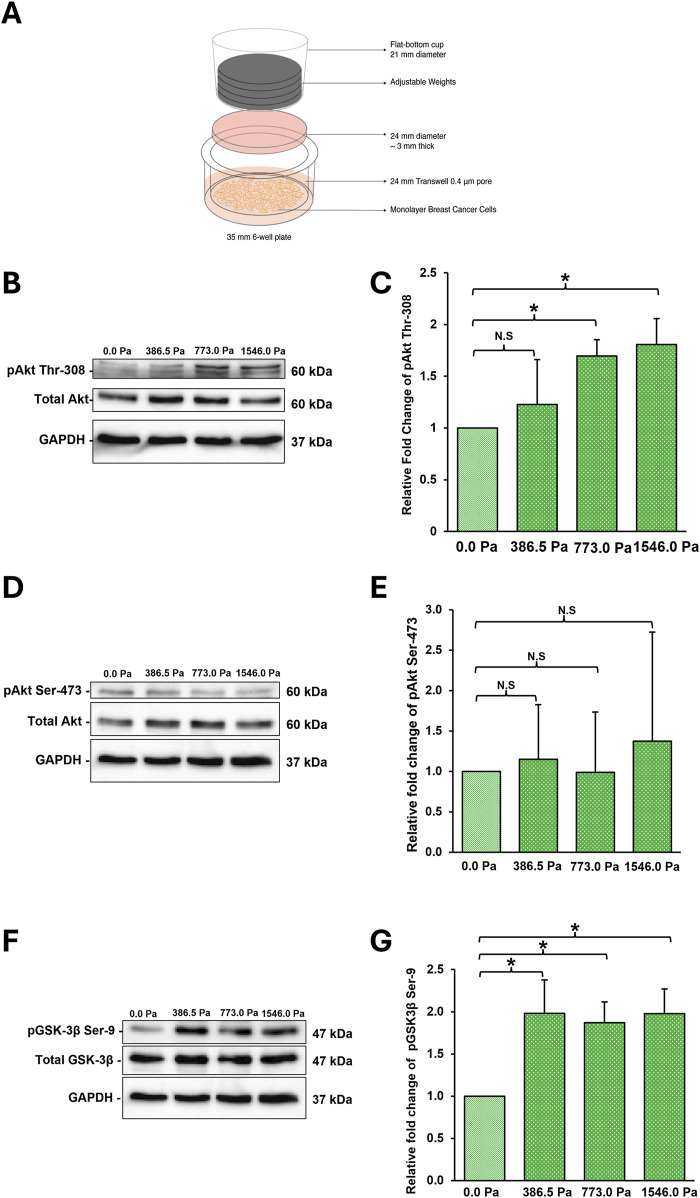
MDA-MB-231 breast cancer cells respond to incremental solid stress compression **(A)** Illustration of 2D *in vitro* compression setup. Briefly, cells are seeded onto the transwell membrane. An agarose disk (2%, ∼3 mm think) is overlayed on top of the cells. Adjustable weights are then placed on the agarose disk. **(B)** Representative western blots highlighting significant changes in phosphorylation of Akt at Thr-308 in MDA-MB-231 cells under 16 h compression at incremental solid stress levels **(C)** Densitometry quantification was done using Image Lab for pAkt Thr-308. Bar graph represents the mean relative fold change as compared to uncompressed across three independent experiments (n = 3). Error bar represents standard deviation. Statistical analysis was performed using 2-tailed student T-test (*) p ≤ 0.05, (**) p ≤ 0.01 and (N.S) Not Significant. **(D)** Representative western blots monitoring phosphorylation of Akt at Ser-473 in MDA-MB-231 cells under 16 h compression at incremental solid stress levels **(E)** Densitometry quantification was done using Image Lab for pAkt Ser-473. Bar graph represents the mean relative fold change as compared to uncompressed across three independent experiments (n = 3). Error bar represents standard deviation. Statistical analysis was performed using 2-tailed student T-test as in **1(C)**. **(F)** Representative western blots highlighting significant changes in phosphorylation of GSK-3β at Ser-9 in MDA-MB-231 cells under 16 h compression at incremental solid stress levels **(G)** Densitometry quantification was done using Image Lab for pGSK-3β at Ser-9. Bar graph represents the mean relative fold change as compared to uncompressed across three independent experiments (n = 3). Error bar represents standard deviation. Statistical analysis was performed using 2-tailed student T-test as in **1(C)**.

We adapted and utilised an established 2D *in vitro* compression setup, as illustrated in Figure 1A, to apply a predefined and uniform pressure on monolayer MDA-MB-231 breast cancer cells (Tse et al., 2012; Kalli et al., 2019). This configuration mimics the exertion of solid stress compression on peripheral cells of an expanding tumour mass, the layer most likely to undergo invasion (Tse et al., 2012). Cells were under compression for 16 h which allows evaluation of steady state responses. This is the same time duration used in previous reports (Tse et al., 2012; Kalli et al., 2019). MDA-MB-231 cells which are frequently used to study metastasis is chosen to model triple-negative breast cancer cells in our experiments (Javed et al., 2023; Conner et al., 2024). Cell viability assay was first conducted to ensure that MDA-MB-231 cells remained viable after the application of incremental solid stress through the 2D *in vitro* setup. Indeed, there were no significant differences between the amounts of viable cells between uncompressed 0.0 Pa condition compared to compressed conditions, 386.5 Pa, 773.0 and 1546.0 Pa (Supplementary Figures S1A,B).

The Akt signalling pathway is implicated and activated through the mechanotransduction of solid stress compression in both cancer and healthy cells (Fernandez-Sanchez et al., 2015; Chen et al., 2017; Kalli et al., 2019). We therefore investigated whether the application of incremental solid stress would activate the Akt/GSK-3β signalling pathway in an incremental manner in breast cancer cells. We found that phosphorylation of Akt at Thr-308 increases with increasing solid stress pressures with cells under 1546.0 Pa showing the greatest increase (Figures 1B,C). No obvious changes in Akt Ser-473 phosphorylation was observed (Figures 1D,E). We also monitored the phosphorylation status of GSK-3β at Ser-9 which is downstream of Akt. Although we observed a significant increase in the phosphorylation of GSK-3β at Ser-9 in compressed cells, this increase does not show positive correlation with increasing solid stress pressure as observed for phospho-Akt (Thr-308). We saw similar levels of phospho-GSK-3β Ser-9 across the three solid stress pressures (Figures 1F,G). Together, these findings suggest that the adapted 2D *in vitro* compression setup was successful at applying solid stress compression and eliciting mechanotransduction responses in MDA-MB-231 cells. We also confirmed that in breast cancer cell type, the Akt/GSK-3β signalling pathway is activated by solid stress compression.

### Incremental solid stress compression impacts on MDA-MB-231 breast cancer cell migration and invasion in a biphasic manner

A wide range of cancer cell types exhibited enhanced migratory phenotype when exposed to solid stress compression (Tse et al., 2012; Fernandez-Sanchez et al., 2015; Chen et al., 2017; Kalli et al., 2022). Aggressive breast cancer cells under solid stress compression have been found to show increased invasion through the activation of Piezo1 which led to increased actin protrusions and matrix metalloproteinase (MMP) activity (Luo et al., 2022). Here, we investigated whether the application of incremental solid stress compression on MDA-MB-231 cells would exhibit a corresponding incremental increase in both cell migration and invasion.

**FIGURE 2:**
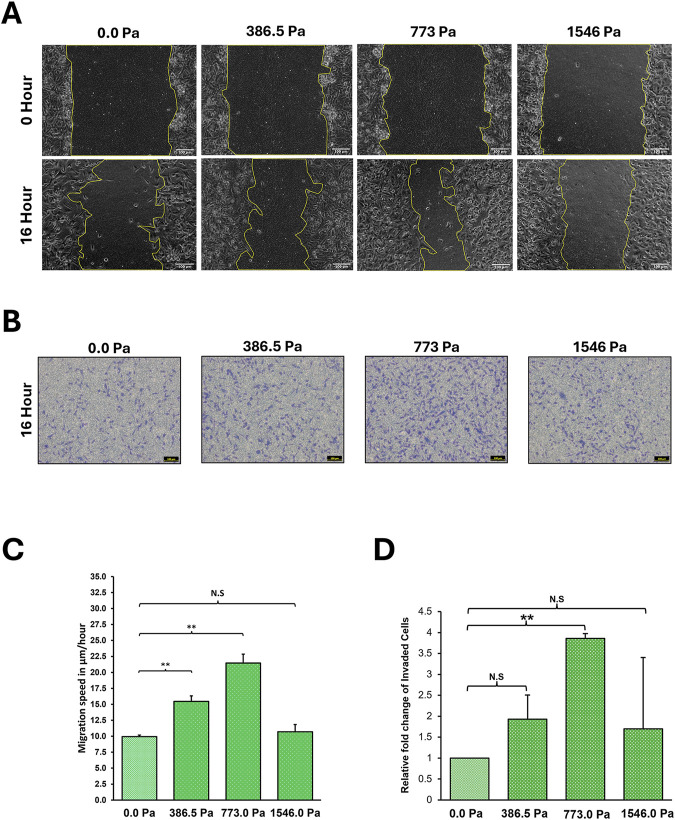
Incremental solid stress compression impacts on MDA-MB-231 cell migration and invasion in a biphasic manner. **(A)** Representative Scratch-Wound Assay images highlighting the changes in MDA-MB-231 cell migration under 16 h compression at various solid stress levels – 386.5 Pa, 773.0 Pa and 1546.0 Pa. Uncompressed cells, 0.0 Pa, were covered with an agarose disk only. Scale bar: 100 μm. **(B)** Representative Invasion Assay images under 16 h compression at various solid stress levels – 386.5 Pa, 773.0 Pa and 1546.0 Pa. Uncompressed cells, 0.0 Pa, were covered with an agarose disk only. Scale bar: 100 μm. **(C)** Analysed by Image Lab, bar graph represents mean speed of wound closure from three independent experiments (n = 3). Error bars represent standard deviation. Statistical analysis was done using 2-tailed student T-test. (*) p ≤ 0.05, (**) p ≤ 0.01 and (N.S) Not Significant. **(D)** Fold changes of number of invaded cells under compression as compared to uncompressed cells. Bar graph represents the mean relative fold change of invaded cells from three independent experiments (n = 3). Error bars represent standard deviation. Statistical analysis was done using 2-tailed student T-test. (*) p ≤ 0.05, (**) p ≤ 0.01 and (N.S) Not Significant.

Interestingly, we found that incremental solid stress enhanced breast cancer cell migration in a biphasic manner with migratory potential increasing up to 773.0 Pa where cells exhibited the greatest migratory capacity (Figures 2A,C). However, under 1546.0 Pa compression, MDA-MB-231 cells exhibited a lower migratory potential compared to the other two compression conditions with levels matching the uncompressed condition, 0.0 Pa (Figures 2A,C). This biphasic trend was also observed in the invasion assay with compression under 773.0 Pa exhibiting the greatest number of invaded cells (Figures 2B,D). Although at 386.5 Pa compression, the mean number of invaded cells is double that of uncompressed cells, statistical analysis indicates the increase as not significant (Figures 2B,D). These findings show that of the three solid stress pressures, 773.0 Pa elicited the greatest increase in both cell migration and invasion. Similar observations were obtained for non-invasive MCF7 breast cancer cells under solid stress compression (Supplementary Figure S2).

### Incremental solid stress compression alters global transcriptome in MDA-MB-231 cells

**FIGURE 3:**
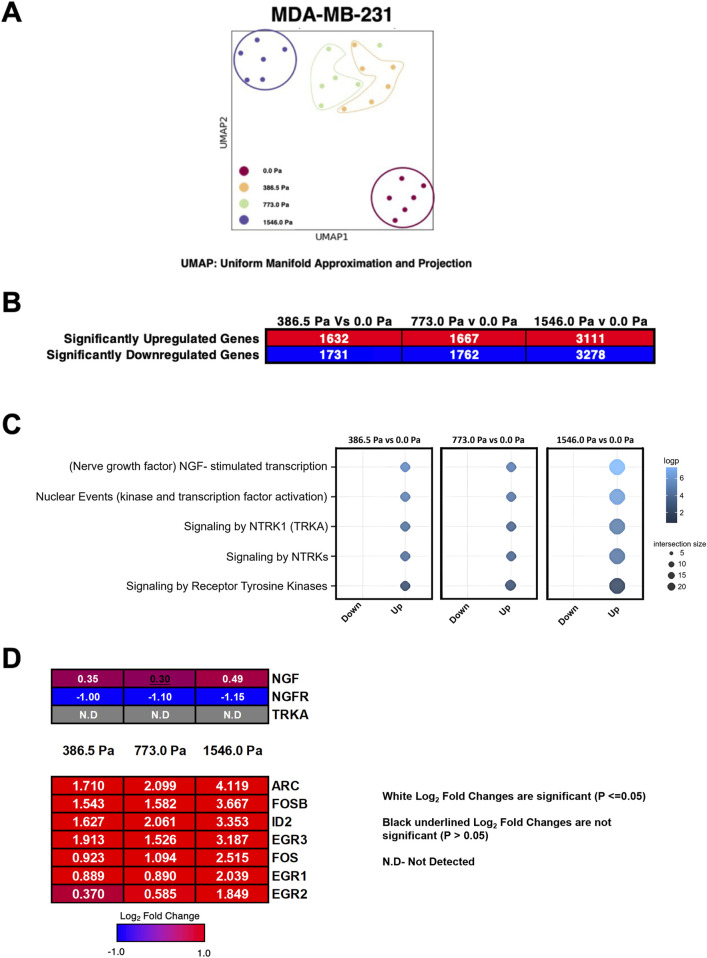
Incremental solid stress compression alters transcriptomes in MDA-MB-231 cells. **(A)** Uniform Manifold Approximation and Projection for Dimension Reduction (UMAP) of six replicates for all four experimental conditions in MDA-MB-231 cells (0.0 Pa, 386.5 Pa, 773.0 Pa and 1546.0 Pa). **(B)** Number of significantly up- and downregulated genes in MDA-MB-231 cells under respective solid stress pressures compared to uncompressed. **(C)** Reactome pathway analysis highlighting five highest ranked pathways. **(D)** Heatmap and Log_2_ fold changes of potential genes involved in the NGF-stimulated transcription pathway. Top panel: NGF (nerve growth factor), NGFR (nerve growth factor receptor), TRKA (tropomyosin receptor kinase A). Bottom panel: ARC (activity-regulated cytoskeleton-associated protein), FOSB (FosB Proto-Oncogene, AP-1 Transcription Factor Subunit), ID2 (Inhibitor of DNA Binding 2), EGR3 (early growth response 3), FOS (Fos Proto-Oncogene, AP-1 Transcription Factor Subunit), EGR1 (Early Growth Response 1), EGR2 (Early Growth Response 2). Log_2_ Fold changes in white fonts are significant (p ≤ 0.05) and Log_2_ fold changes in black underlined fonts are not significant (p > 0.05).

Transcriptomic studies of cancer cells under compression have discovered differentially expressed genes pertinent to cancer progression (Demou, 2010; Chen et al., 2017; Kalli et al., 2019; Kalli et al., 2022). Pancreatic cancer cells adopted a more aggressive metastatic phenotype through the upregulation of Growth Differentiation Factor 15 (GDF15) (Kalli et al., 2019). Compression on breast cancer cells resulted in downregulation of miR-9 which impacts on VEGF (Kim et al., 2017). Meanwhile, compression on breast cancer associated fibroblasts induced upregulation of metabolic genes (Kim et al., 2019). In this study, we investigated the global transcriptome alterations in MDA-MB-231 cells under incremental solid stress compression to further identify mechanosensitive genes responsible for the enhanced aggressive phenotype especially in cells under compression at 773.0 Pa. RNA Sequencing (RNA-Seq) was performed on six biological replicates and their respective data clusters were presented using the uniform manifold approximation and projection for dimension reduction (UMAP). This non-linear dimensionality reduction tool shows that uncompressed (0.0 Pa) data from six replicates cluster together and appear well separated from the six replicates of respective compressed data clusters, suggesting that the two groups of datasets between compressed and uncompressed (in particular, 1546.0 Pa vs. 0.0 Pa) are distinct and different (Figure 3A). We found a total of 1632, 1667 and 3111 significantly upregulated genes in cells under incremental solid stress pressure of 386.5 Pa, 773.0 Pa and 1546.0 Pa, respectively (Figure 3B). Meanwhile, 1731, 1762, and 3278 significantly downregulated genes were found in cells under compressive pressures of 386.5 Pa, 773.0 Pa and 1546.0 Pa, respectively (Figure 3B). These findings suggest that solid stress compression exerts significant transcriptome alterations in MDA-MB-231 breast cancer cells.

The Reactome pathway analysis (www.reactome.org) was utilised to provide insights on pathway enrichment of the differentially regulated genes. Here we show the top 5 enriched pathways in cells under incremental solid stress compression with the Nerve Growth Factor (NGF)-stimulated transcription pathway ranked the highest amongst the pathways (Figure 3C). The heatmap illustrates genes potentially involved in the NGF-stimulated transcription pathway with their respective Log_2_ fold changes (Figure 3D). NGF, a member of the neurotrophins family, plays important roles in both survival and differentiation of neurons. NGF has also been implicated in the progression of human cancers especially in aggressive breast cancers (Molloy et al., 2011; Di Donato et al., 2021; Bruno et al., 2022). NGF exerts its function by binding to two unrelated receptors: Nerve Growth Factor Receptor (NGFR), also known as p75NTR, and Tyrosine Kinase A (TrkA) (Di Donato et al., 2021). Downstream gene targets of the NRF pathway such as Activity-Regulated Cytoskeleton-Associated (ARC) and FBJ murine osteosarcoma viral oncogene homolog B (FOSB) were significantly upregulated across the three solid stress pressures (Figure 3D). Although the Reactome analysis suggests that the NGF-stimulated transcription pathway was enriched, the Log_2_ fold changes of the NGF gene itself was only minimally upregulated under incremental solid stress with cells under 773.0 Pa showing no significant increase in NGF (Figure 3D). Furthermore, the NGF receptor (NGFR), which has been implicated in breast cancer invasion and metastasis (Bruno et al., 2022), was significantly downregulated across the three solid stress pressures (Figure 3D). The other NGF receptor, TrkA, also involved in breast cancer metastasis (Bruno et al., 2022), was not detected in our RNA-Seq analysis (Figure 3D). Whilst we observed higher NGF downstream gene transcripts, the minimal Log_2_ fold increase of NGF and the stark downregulation NGFR persuaded us to look for other significantly upregulated genes and pathways that are biologically relevant to breast cancer metastasis.

**FIGURE 4:**
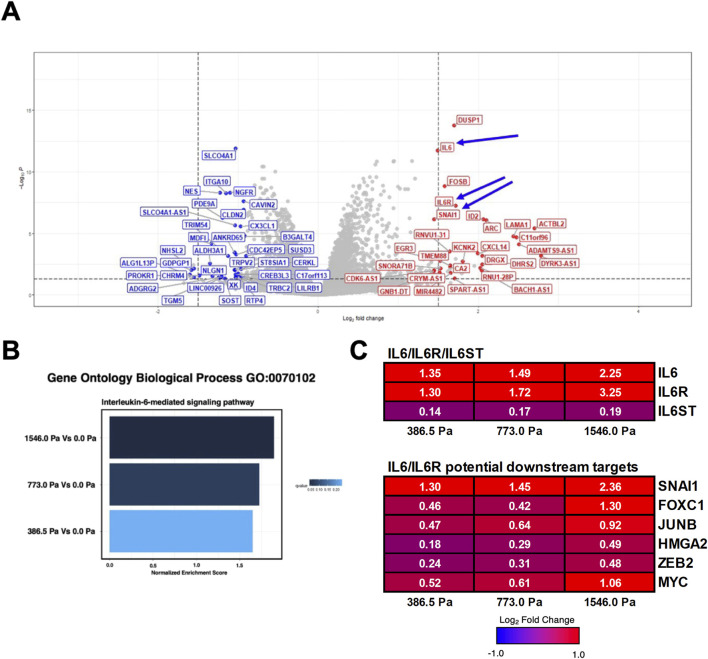
Solid stress compression implicates the activation of Interleukin-6 (IL-6) signalling pathway. **(A)** Representative volcano plot highlighting the top ∼40 upregulated (in red) and ∼40 downregulated (in blue) genes in MDA-MB-231 cells under 773.0 Pa in comparison to uncompressed cells (0.0 Pa). Arrows indicate the upregulation of IL-6, IL-6 Receptor and its potential downstream gene target, SNAI1. Respective volcano plots and table listing the top 40 upregulated and downregulated genes for all three incremental solid stress pressures are in Supplementary Figures 3–5. **(B)** Normalised Enrichment Score (NES) of IL-6 mediated signalling pathway in MDA-MB-231 cells under incremental solid stress pressures. **(C)** Heatmap and Log_2_ fold changes of potential genes involved in IL-6 signalling pathway. Top panel: IL6 (interleukin 6), IL6R (interleukin 6 receptor), IL6ST (interleukin 6 signal transducer or Glycoprotein 130). Bottom panel: SNAI1: snail family transcription repressor 1; FOXC1: forkhead box protein C1; JUNB: JunB proto-oncogene; HMGA2: high-mobility group AT-hook 3; ZEB2: zinc finger E-box binding homeobox 2. Log_2_ Fold changes in white fonts are significant (p ≤ 0.05).

We next examined the volcano plots (Supplementary Figures S3–5) to identify mechanosensitive genes based on the following criteria: 1) genes that are significantly upregulated across the three solid stress pressures and 2) genes that are biologically relevant to cell migration and metastasis. Interestingly, we found that Interleukin-6 (IL-6) and its receptor IL-6R were significantly upregulated (Figures 4A,C). Given the prominence of IL-6 in breast cancer migration, invasion and metastasis (Zhou et al., 2017; Hou et al., 2018; Johnson et al., 2018; Cho et al., 2020; Chen et al., 2022; Manore et al., 2022; Radharani et al., 2022), we evaluated the Normalised Enrichment Score (NES) of the IL-6 signalling in our data set. We found that the IL-6 signalling pathway is upregulated with increasing order of solid stress pressure (Figures 4B,C). Specifically, MDA-MB-231 cells under 386.5 Pa, 773.0 Pa and 1546.0 Pa exhibited 1.35, 1.49, and 2.25 times Log_2_ fold increase in IL-6 transcripts, respectively compared to uncompressed cells (Figure 4C). Similarly, under 386.5 Pa, 773.0 Pa and 1546.0 Pa, MDA-MB-231 cells exhibited 1.30, 1.72 and 3.25 times log_2_ fold increase in IL-6R transcripts, respectively compared to uncompressed cells (Figure 4C). Given the probable involvement of IL-6 signalling pathway in response to solid stress compression, we further investigated some of the key downstream gene targets. We found that snail family transcriptional repressor 1 (SNAI1), a master regulator of epithelial to mesenchymal transition (Manore et al., 2022), was significantly upregulated at 1.30, 1.45 and 2.36 times (Log_2_ fold) in cells under 386.5 Pa, 773.0 Pa and 1546.0 Pa, respectively compared to uncompressed cells (Figure 4C). MYC, another downstream gene target of IL-6 signalling pathway (Manore et al., 2022), was also upregulated (Figure 4C). Both genes have been implicated in breast cancer progression and metastasis. The upregulation of IL-6/IL-6R and its potential downstream gene targets including SNAI1 and MYC suggests that the IL-6 signalling pathway could be activated in response to solid stress compression.

**FIGURE 5:**
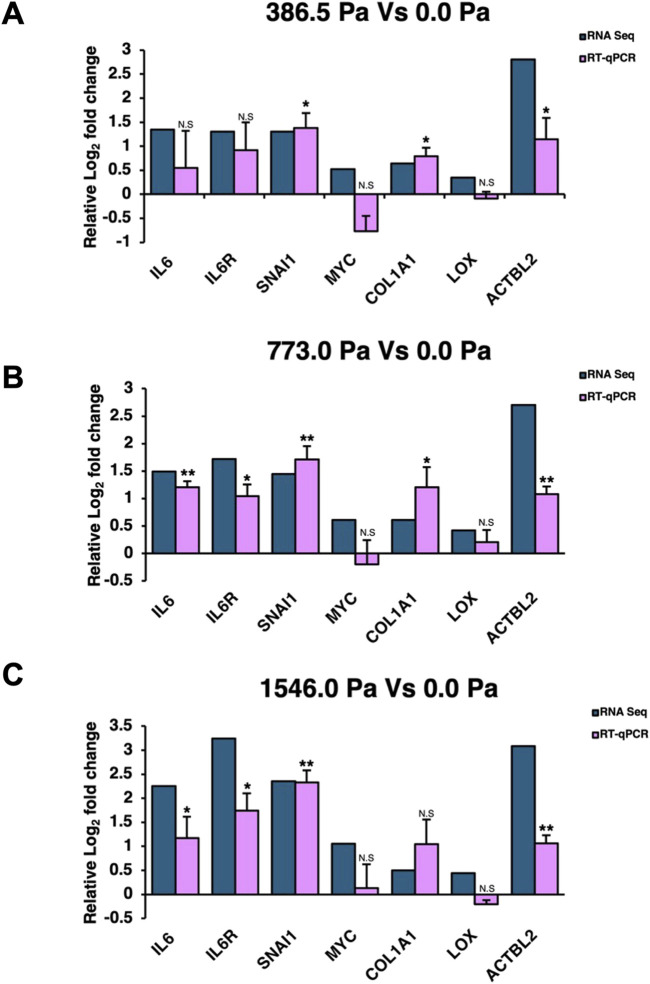
Validation of RNA-Seq gene targets. Relative Log_2_ fold changes of selected gene targets from RNA-Seq and reverse transcription quantitative polymerase chain reaction (RT-qPCR) in **(A)** 386.5 Pa Vs 0.0 Pa data set, **(B)** 773.0 Pa Vs 0.0 Pa data set., and **(C)** 1546.0 Pa Vs 0.0 Pa data set. RT-qPCR data are from three independent repeats (n = 3) with error bars denoting standard deviation. Blue bars represent Log_2_ fold change of RNA-Seq and pink bars represent RT-qPCR Log_2_ fold changes.

Reverse transcription-quantitative polymerase chain reaction (RT-qPCR) was performed to validate the transcript levels of several differentially expressed gene targets from the RNA-Seq analysis. Both IL-6 and IL-6R together with their downstream gene targets, SNAI1 and MYC, were chosen for validation. Collagen Type I Alpha 1 Chain (COL1A1) and Lysyl Oxidase (LOX) were included as they were upregulated across the three solid stress compression (Supplementary Figure S6A) and are important in modulating the tumour microenvironment as well as promoting cancer migration (Payne et al., 2005; Liu et al., 2018). In addition, Actin Beta Like 2 (ACTBL2), a novel actin isoform, was also validated because of the high Log_2_ Fold changes in cells across the three solid stress pressures and its reported role in cancer cell. All except MYC and LOX could be validated by RT-qPCR (Figures 5A–C), although we do observe increasing levels of MYC with increasing compression pressures using RT-qPCR. IL-6, IL-6R and their downstream gene target, SNAI1, were found to be significantly upregulated in our RT-qPCR data especially in MDA-MB-231 cells under 773.0 Pa (Figure 5B).

### Incremental solid stress compression upregulates the protein levels of IL-6 and SNAI1 as well as secretion of IL-6

Both RNA-Seq and RT-qPCR data suggest the upregulation of IL-6 and its potential downstream gene target, SNAI1, in MDA-MB-231 cells under incremental solid stress compression (Figures 4, 5). We also found that MDA-MB-231 cells under incremental solid stress exhibited enhanced cell migration and invasion in a biphasic manner which peaked at 773.0 Pa compression (Figure 2). We next proceeded to determine the protein levels of IL-6 and SNAI1 to ascertain if the aggressive phenotypic changes observed (Figure 2) could be related to IL-6 signalling.

**FIGURE 6:**
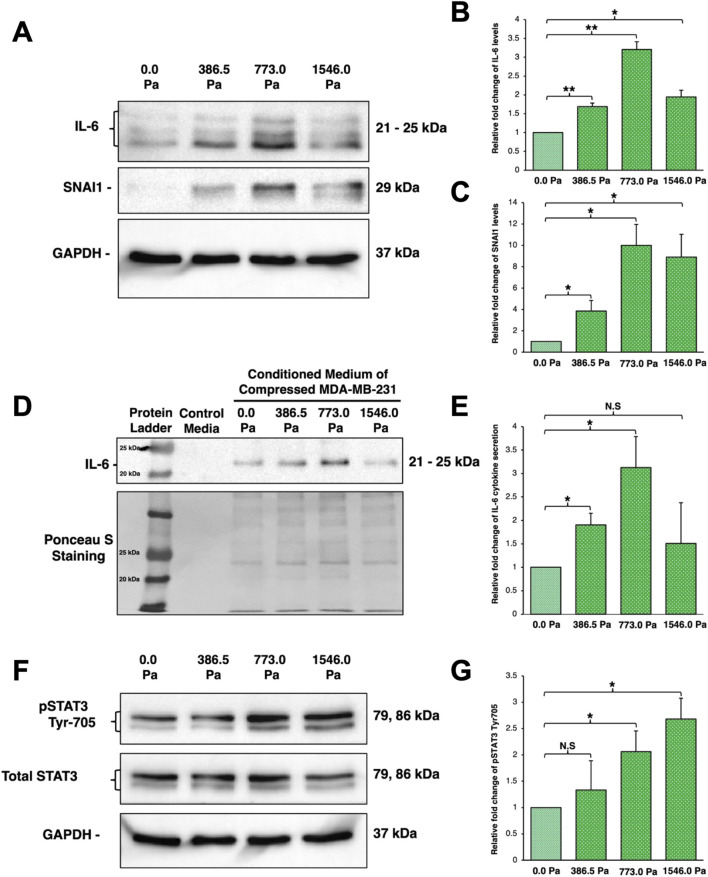
Incremental solid stress compression upregulates protein levels of IL-6 and SNAI1 as well as secretion of IL-6 in a biphasic manner **(A)** Representative western blots of cell lysates harvested from MDA-MB-231 cells under 16 h of incremental solid stress compression using IL-6 and SNAI1 antibodies. GAPDH was used as loading control **(B)** Densitometry quantification of IL-6 protein levels using Image Lab. Bar graph represents the mean relative fold changes compared to uncompressed across three independent experiments (n = 3). Error bars represent standard deviation. Statistical analysis was performed using 2-tailed student T-test (*) p ≤ 0.05, (**) p ≤ 0.01 **(C)** Densitometry quantification of SNAI1 protein level using Image Lab. Bar graph represents the mean relative fold changes compared to uncompressed across three independent experiments (n = 3). Error bars represent standard deviation. Statistical analysis was performed using 2-tailed student T-test, (*) p ≤ 0.05, (**) p ≤ 0.01. **(D)** (Top) Representative Western blot of conditioned media harvested after 16 h of incremental solid stress compression and probed with anti-IL-6 antibody. (Bottom) Representative Ponceau S Staining highlighting equal protein loading of the conditioned media. **(E)** Densitometry quantification of IL-6 cytokine secretion using Image Lab. Bar graph represents the mean relative fold changes compared to uncompressed across three independent experiments (n = 3). Error bars represent standard deviation. Statistical analysis was performed using 2-tailed student T-test, (*) p ≤ 0.05 and (N.S) Not Significant. **(F)** Representative Western blot image using anti-phospho-STAT3 (pSTAT3) at Tyr-705 antibody for MDA-MB-231 cells under 16 h of incremental solid stress compression. **(G)** Densitometry quantification of pSTAT3 using Image Lab. Bar graph represents the mean relative fold changes compared to uncompressed across three independent experiments (n = 3). Error bars represent standard deviation. Statistical analysis was performed using 2-tailed student T-test, (*) p ≤ 0.05 and (N.S) Not Significant.

Indeed, we found that MDA-MB-231 cells under incremental solid stress compression exhibited a significant increase in IL-6 and SNAI1 protein levels (Figures 6A–C). More specifically, IL-6 protein levels follow a biphasic trend with peak levels observed at 773.0 Pa compression (Figures 6A,B). Interestingly, under the highest compressive pressure of 1546.0 Pa, IL-6 protein levels did not increase further but found to be reduced compared to those under 773.0 Pa (Figures 6A,B). Similarly, SNAI1 protein levels were also significantly upregulated in a similar biphasic trend with cells under 773.0 Pa showing the greatest increase (Figures 6A,C). The protein levels of IL-6 and SNAI1 across the three compressive pressures appear to mirror the biphasic trend observed for cell migration and invasion (Figures 2, 6A–C).

Clinical studies on breast cancer patients found elevated IL-6 levels at both the edges of breast tumour masses and in patient’s sera as a consequence of overexpression and secretion by breast cancer cells (Hou et al., 2018; Johnson et al., 2018; Chen et al., 2022; Manore et al., 2022). Secreted IL-6 cytokines would then function in autocrine and paracrine manners to exacerbate breast cancer progression. Our observations thus far led us to hypothesise that compression on breast cancer cells leads to secretion of IL-6 cytokine in a similar biphasic trend to exert pro-metastatic function in autocrine and paracrine signalling (Figures 2, 6A–C). To test this hypothesis, Western blot was performed on concentrated conditioned media collected after compression. We found incremental solid stress compression elicited greater IL-6 cytokine secretion in a similar biphasic trend with cells under 773.0 Pa exhibiting the greatest amount of IL-6 cytokine secretion (Figures 6D,E). Cells under 386.5 Pa also showed a significant increase in IL-6 protein secretion whilst cells under the highest pressure of 1546.0 Pa showed no significant increase (Figures 6D,E). 

We next investigated if IL-6 signalling pathway is activated. The key signal transducer of the IL-6 signalling pathway is Signal Transducer and Activator of Transcription-3 (STAT3). Binding of IL-6 to its receptors leads to phosphorylation and activation of STAT3 by JAK kinase. Upon activation, STAT3 homo-dimerises and translocates into the nucleus to upregulate transcription of target genes such as SNAI1 (Johnson et al., 2018; Abaurrea et al., 2021; Chen et al., 2022; Manore et al., 2022). We thus monitored the phosphorylation levels of STAT3 at Tyr-705 in MDA-MB-231 cells under incremental solid stress. In contrast to the IL-6 secretion trend, we found that phosphorylation of STAT3 at Tyr-705 increased with increasing solid stress pressure (Figures 6F,G). Cells under 773.0 Pa and 1546.0 Pa exhibited a significant increase in pSTAT3 levels with cells under 1546.0 Pa exhibiting the highest levels of pSTAT3 (Figures 6F,G).

### Interleukin-6 knockdown attenuates solid stress-induced cell migration

**FIGURE 7:**
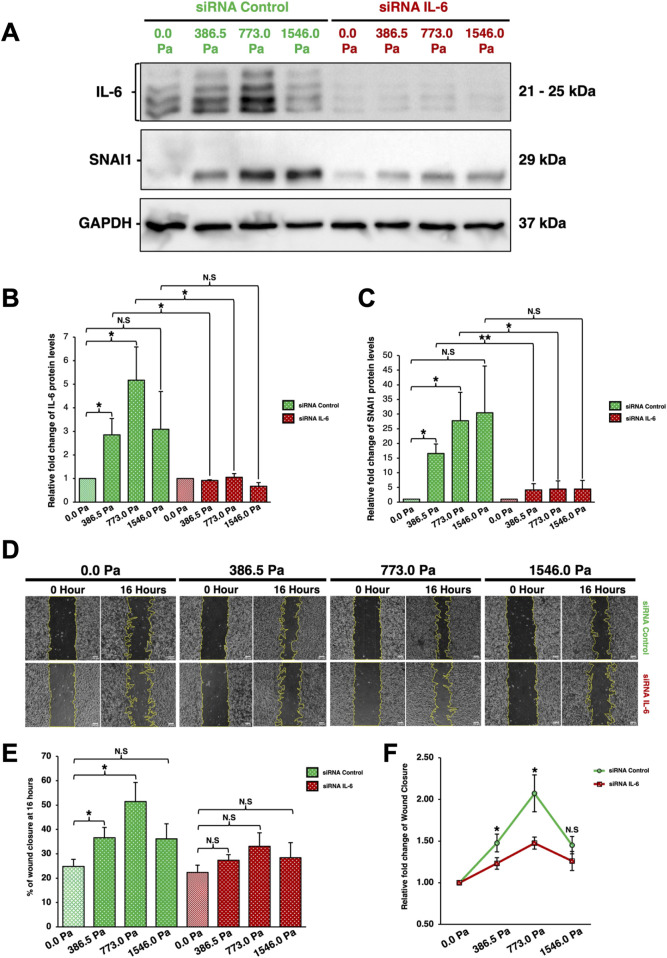
Interleukin-6 knockdown through siRNA-IL-6 downregulates SNAI1 protein and attenuates solid stress-induced cancer cell migration **(A)** Cells were transfected with control and IL-6 siRNAs. Different compressive stress was then applied. After 16 h of compression, cell lysates were harvested and analysed by Western blotting using the antibodies indicated. Representative western blots of cells under 16 h of incremental solid stress compression are shown here. **(B)** Densitometry quantification of IL-6 using Image Lab. Bar graph represents the mean relative fold changes compared to uncompressed across three independent experiments (n = 3). Error bars represent standard deviation. Statistical analysis was performed using 2-tailed student T-test, (*) p ≤ 0.05, (**) p ≤ 0.01 and (N.S) Not Significant. **(C)** Densitometry quantification of SNAI1 using Image Lab. Bar graph represents the mean relative fold changes compared to uncompressed across three independent experiments (n = 3). Error bars represent standard deviation. Statistical analysis was performed using 2-tailed student T-test, (*) p ≤ 0.05, (**) p ≤ 0.01 and (N.S) Not Significant. **(D)** Representative Scratch-Wound Assay images highlighting cell migration alterations between cells transfected with siRNA-Control or siRNA-IL-6 under 16 h compression at various solid stress levels – 386.5 Pa, 773.0 Pa and 1546.0 Pa. Uncompressed cells, 0.0 Pa, were covered with an agarose disk only. Scale bar: 100 μm. **(E)** Analysis was done using Image Lab. Bar graph represents mean percentages of wound closure from three independent experiments (n = 3). Error bars represent standard deviation. Statistical analysis was performed using 2-tailed student T-test. (*) p ≤ 0.05, and (N.S) Not Significant. **(F)** Relative fold change of wound closure comparison between cells transfected with either siRNA-Control or siRNA-IL-6. Error bars represent standard deviation. Statistical analysis was performed using 2-tailed student T-test. (*) p ≤ 0.05, and (N.S) Not Significant.

To elucidate IL-6’s role in solid stress-induced cell migration, small interfering RNA (siRNA) was utilised to knockdown IL-6 prior to migration assays. Western blot analysis was employed to validate the levels of different proteins after IL-6 knockdown. After 16 h of compression, MDA-MB-231 cells transfected with siRNA-Control exhibited a similar biphasic trend in IL-6 levels as previously observed (Figures 6, 7A,B). Meanwhile, cells transfected with siRNA-Control exhibited an increasing trend in SNAI1 protein levels under incremental solid stress compression (Figures 7A,C). As expected, MDA-MB-231 cells transfected with siRNA-IL-6 exhibited a substantial decrease in IL-6 levels (Figures 7A,B). Compressed breast cancer cells transfected with siRNA-IL-6 exhibited a sharp decrease in SNAI1 protein levels compared to cells transfected with siRNA-Control (Figures 7A,C). The overall SNAI1 levels are reduced in cells transfected with siRNA-IL-6. Although the levels of SNAI1 protein are slightly higher in compressed cells compared to the uncompressed cells (Figures 7A,C), incremental solid stress-induced upregulation of SNAI1 (Figures 6A,C) was not observed when IL-6 was knocked down (Figures 7A,C).

We found that the IL-6 knockdown impaired solid stress-induced cell migration in MDA-MB-231 cells (Figures 7D–F). Cells transfected with siRNA-Control exhibited enhanced cell migration in a biphasic pattern under incremental solid stress compression, similar to compressed cells in Figures 2A,C, with cells under 773.0 Pa having the greatest migratory capacity followed by cells under 386.5 Pa (Figures 7D–F). In contrast, upon IL-6 knockdown, the migratory capacity of compressed cells across all three solid stress pressures showed no significant changes when compared to the uncompressed cells (Figures 7D–F). The difference in relative fold change of wound closure between the compressed and uncompressed cells is the most prominent for cells under 773.0 Pa (Figure 7F). Our data demonstrate that IL-6 knockdown attenuated solid stress-induced cell migration, suggesting that the observed upregulation of IL-6 protein and secretion levels in compressed breast cancer cells could play an underlying role in solid stress-induced cancer cell migration (Figures 2, 6, 7).

## Discussion

Throughout tumour growth and progression, tumour masses are exposed to a range of biomechanical stimuli from the tumour microenvironment ranging from stiffening of the extracellular matrices to fluid shear stress and solid stress compression. Various studies have shown that these biomechanical stimuli can elicit tumorigenesis, exacerbate cancer progression and metastasis as well as enhance resistance to chemotherapy (Levental et al., 2009; Schrader et al., 2011; Lang et al., 2015; Yang et al., 2016; Lee et al., 2017; Joyce et al., 2018; Lee et al., 2018; Northcott et al., 2018; Dong et al., 2019). Solid stress compression, particularly experienced by peripheral cancer cells of the tumour mass, has been implicated in supporting tumorigenesis, promoting epithelial to mesenchymal transition, inducing cancer stemness and contributing to aggressive and metastatic phenotypes (Demou, 2010; Tse et al., 2012; Fernandez-Sanchez et al., 2015; Chen et al., 2017; Kalli et al., 2019; Kalli et al., 2022; Luo et al., 2022). In this study, we investigated the global transcriptomic and metastatic phenotype alterations in MDA-MB-231 breast cancer cells under incremental solid stress compression ranging from 386.5 Pa, 773.0 Pa and 1546.0 Pa, using an established 2D *in vitro* compression setup. In agreement with previous solid stress studies, we show that MDA-MB-231 cells under incremental solid stress compression exhibited higher phosphorylation levels of both Akt and GSK-3β (Figures 1B,C) (Fernandez-Sanchez et al., 2015; Chen et al., 2017; Kalli et al., 2019). Although we observed increased GSK-3β phosphorylation across the three compressive pressures applied, we do not see a stepwise increase corresponding to the incremental compressive stress. Our experimental data suggest that solid stress compression at 386.5 Pa is sufficient to result in observable GSK-3β Ser-9 phosphorylation. We are not able to decipher why and how AKT phosphorylation levels at Thr 308 and GSK-3β phosphorylation at Ser 9 do not show similar trends with respect to incremental solid stress compression. Apart from the regulation by kinases, both AKT and GSK-3β are also regulated by phosphatases, including PP1 and PP2A (Hernandez et al., 2010; Xiao et al., 2010). We expect interplay and crosstalk between the kinases and phosphatases which will eventually determine the levels of phospho-AKT and phospho-GSK-3β observed after 16 h of compression. Currently, we do not know if compression can directly lead to phosphorylation of AKT in breast cancer cells. It has been reported that phosphorylated AKT is observed in MIA PaCa-2 cells under compression. Treatment of PI3K inhibitor blocked AKT phosphorylation and stress-induced motility, suggesting PI3K is the upstream kinase which is activated by compression (Kalli et al., 2019).

We found that MDA-MB-231 cells under incremental solid stress compression exhibited enhanced migratory potential in a biphasic manner with cells under 773.0 Pa demonstrating the greatest migratory capacity (Figures 2A,C). This biphasic migratory capacity is not exclusive to solid stress compression as it was also observed in cells exposed to increasing matrix stiffness and fluid shear stress (Lee et al., 2017; Yip et al., 2021). When plated on increasing substrate rigidity ranging from 6 kPa, 14 kPa to 31 kPa, NIH3T3 cells exhibited a similar biphasic trend with cells on intermediate stiffness (14 kPa) exhibiting the greatest migratory capacity (Yip et al., 2021). Likewise, when exposed to increasing fluid shear stress from 0.5 dyne/cm^2^, 1.0 dyne/cm^2^ to 5.0 dyne/cm^2^, human prostate cancer cells exhibited a similar biphasic trend with cells under the intermediary stress of 1.0 dyne/cm^2^ having the greatest cell migration capacity (Lee et al., 2017). In invasion assays, we found that compressed MDA-MB-231 cells exhibit a similar biphasic trend with cells under 773.0 Pa exhibiting the greatest number of invaded cells (Figures 2B,D). It is unclear why intermediary stresses/stiffness are optimal and favourable for enhanced migratory phenotype whilst higher stresses/stiffness appears to reverse this aggressive phenotype. Our current observations suggest that the pathophysiologically-relevant breast tumour microenvironment pressure (773.0 Pa) elicited the most aggressive metastatic phenotype.

From RNA sequencing, RT-qPCR and Western blot analysis, we found that IL-6, its receptor IL-6R, and SNAI1 were significantly upregulated under the three solid stress pressures (Figures 4–6). We observed that IL6 and SNAI1 protein levels peaked at 773.0 Pa while the transcript levels of both IL6 and SNAI1 are highest at 1546.0 Pa. It is noteworthy that transcript levels do not always corelate with protein levels. Many studies have reported that transcript levels are not sufficient to predict protein levels. However, at steady state conditions, protein levels are in general determined by transcript levels and translation efficiency (Liu et al., 2016; Srivastava et al., 2022). The discrepancy between protein and transcript levels is attributed to sample differences and techniques used for both transcriptome/mRNA and proteome/protein analysis. Post-transcription regulation and protein-protein interaction also affect the transcript and protein stability and levels. In our case, it is difficult to determine the underlying mechanism. It could be due to mechanosensitive regulation of gene transcription and/or protein translation/stability. Interestingly, IL-6 cytokine was secreted in a similar biphasic pattern with cells under 773.0 Pa secreting the highest amounts of IL-6 cytokines (Figures 6D,E). This finding offers a prospective insight for the elevated IL-6 cytokines levels observed in both the sera of breast cancer patients as well as at the edges of breast tumour masses (Hou et al., 2018; Johnson et al., 2018; Chen et al., 2022; Manore et al., 2022). pSTAT3 at Tyr-705 levels increased with increasing solid stress pressure, a trend that does not match the biphasic trend of IL-6 secretion (Figures 6F,G). This discrepancy could be associated with STAT3’s role as a signal transducer for several signalling pathways ranging from epidermal growth factors to other cytokines apart from IL-6 (Ma et al., 2020). It is possible that compressed MDA-MB-231 cells could secrete epidermal growth factors and other cytokines such Interleukin-8 (IL-8, also known as CXCL8) that could also contribute to the increasing levels of pSTAT3 observed (Martinez-Carpio et al., 1999; Todorovic-Rakovic and Milovanovic, 2013; Ma et al., 2020). Coincidentally, from our RNA-Seq data, we found increased IL-8 (Supplementary Figure S6B) in cells subjected to compression at 773.0 Pa (log_2_ fold change 0.48) and 1546.0 Pa (log_2_ fold change 1.99). In addition, we found increased NGF signalling (Figures 3C,D) at 1534.0 Pa compressive pressure, and NGF receptor TrkA has been shown to phosphorylate STAT3 at Tyr-705 (Regua et al., 2021), it is possible that downstream signalling of NGF/TrkA may also contribute to high phospho-STAT3 Tyr-705 levels at 1534.0 Pa.

Silencing IL-6 led to reduction in migratory potential for cells under compressive stress (Figures 7D–F), suggesting that solid stress-induced cancer cell migration in compressed MDA-MB-231 cells could be dependent on IL-6 levels (Figures 7D–F). IL-6 knockdown also led to reduced SNAI1 protein levels in compressed MDA-MB-231 cells (Figures 7A–C). Therefore, we hypothesise that the upregulation of SNAI1 protein levels observed in compressed breast cancer cells could be due to the solid stress-induced upregulation of IL-6 (Figures 7A–C). It has been reported that in the presence of IL-6 secreted from adipocytes, breast cancer cells (MDA-MB-468 and MCF-7) exhibited enhanced migratory and invasive capacity as a consequence of IL-6 protein and SNAI1 transcription upregulation (Gyamfi et al., 2018). Inhibition of IL-6 signalling pathway through neutralising IL-6 antibody and siRNA found both breast cancer cell lines exhibited decreased migratory and invasive potential with reduced SNAI1 transcription (Gyamfi et al., 2018). In head and neck tumour cells (CAL27), exposure to IL-6 cytokines upregulated SNAI1 protein levels and enhanced cancer cell migration (Yadav et al., 2011). *In vivo* experiments in mice show that xenografts of IL-6-overexpressing CAL27 resulted in enhanced metastatic potential as indicated by increased lymph node and lung metastasis (Yadav et al., 2011). Here, we found IL-6’s involvement in solid stress-induced metastatic phenotype alterations of breast cancer cells.

**FIGURE 8:**
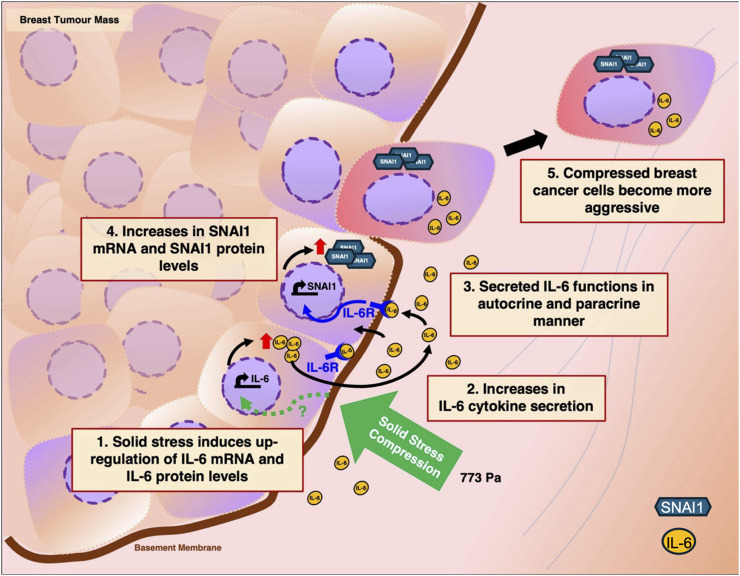
Proposed working model on how solid stress compression enhances the metastatic phenotype of breast cancer cells through the upregulation of Interleukin-6. 1. Rapid expansion of tumour mass results in the exertion of solid stress compression on breast cancer cells, in particular the peripheral layer of cells. Consequently, mechanotransduction of solid stress compression results in the upregulation of IL-6 mRNA and IL-6 protein levels through a pathway still not known (green dotted arrow). 2. The compressed breast cancer cells secrete more IL-6 cytokines into the tumour microenvironment. 3. Secreted IL-6 cytokines then function in autocrine and paracrine manners. 4. Increased IL-6 secretion results in the activation of IL-6 receptors which in turn leads to upregulation of SNAI1 mRNA and protein levels. 5. Compressed breast cancer cells with elevated IL-6 and SNAI1 proteins gain a more aggressive phenotype, migrate away from primary tumour site and invade neighbouring tissues.

Together, we propose a working model as illustrated in Figure 8. As a breast tumour mass expands within the body, peripheral cells of the tumour experience higher solid stress compression. This results in the upregulation of IL-6 transcripts and proteins through a pathway still not known (Green dotted arrow, Figure 8). Consequently, these compressed breast cancer cells secrete more IL-6 cytokines into the tumour microenvironment where it then functions in autocrine and paracrine manners to upregulate downstream gene targets of IL-6 such as SNAI1. SNAI1 is a zinc finger transcription factor that is associated with EMT. It can suppress the expression of E-cadherin and increase expression of mesenchymal markers such as vimentin and fibronectin (Kaufhold and Bonavida, 2014). SNAI1 overexpression has also been implicated in the induction of migration and invasion (Singh et al., 2021). As a result of IL-6 and SNAI1 upregulation, compressed breast cancer cells exhibit a more aggressive metastatic phenotype and are more likely to undergo migration and invasion to distal sites.

At the moment, it is unclear how mechanotransduction of solid stress leads to the upregulation of IL-6 protein levels. It has been reported that in cardiac fibroblast cells, exposure to mechanical stress leads to the opening of Piezo1, a mechanosensitive ion channel, which in turn results in the upregulation of IL-6 through the p38α MAPK signalling pathway (Blythe et al., 2019). Breast cancer cells under solid stress compression also exhibited enhanced invasion through Piezo1 activation (Luo et al., 2022). Therefore, it is tempting to postulate that the observed IL-6 increase in our study could be due to Piezo1 activation. Interestingly, we did not find increase in Piezo1 transcript levels but found increase in another mechanosensitive channel, KCNK2 (also known as TREK1) in our RNA-Seq dataset (Supplementary Figure S6C). Further investigation is needed to understand why less IL-6 proteins and IL-6 secretion were found in MDA-MB-231 cells under compression of 1546.0 Pa when compared to cells under 773.0 Pa (Figures 6, 7). We hypothesise that under 1546.0 Pa compressive pressure, compressed breast cancer cells might be in a stress-induced translational control state where global mRNA translation is reduced and hence the observed reduction in protein levels of IL-6 and SNAI1. Stress-induced translational control has been reported for cells under hypoxia, nutritional and genotoxic stress (Leprivier et al., 2015). Similarly, mechanical stress can impact the translational machinery through mechanical signalling pathways and the cytoskeleton (Goransson and Stromblad, 2024). In addition, mechanical cues could also affect protein stability through the modulation of protein degradation by affecting normal activities of ubiquitin ligases and proteasomes (Goransson and Stromblad, 2024). In summary, this study shows that solid stress compression can lead to upregulation of IL-6 levels and signalling which in turn contribute towards metastatic behaviour of breast cancer cells. We also found that incremental solid stress compression leads to the activation of the Akt/GSK-3β pathway. GSK-3β can further impact on β-catenin signalling. It has been found that β-catenin can inhibit IL-6 expression (Edara et al., 2020; Robinson et al., 2020), while IL-6 can activate the Wnt/β-catenin signalling pathway (Zi et al., 2023). Closer to our current experimental context, it has been reported that neither compression alone nor treatment with IL-6 alone results in β-catenin translocation to the nucleus (Chen et al., 2017). However, combination of compression and IL-6 treatment leads to β-catenin translocation. Therefore, the crosstalk between the Akt/GSK-3β pathway and the IL-6 signalling pathway warrants further study.

## Materials and methods

### Overview of experimental workflow

MDA-MB-231 breast cancer cells were initially seeded at a density of 4 × 10^5^ cells/Transwell in DMEM4500 10% FBS and allowed to reach near confluency prior to a media change. Media for MDA-MB-231 cells were then changed to DMEM4500 0% FBS. After 24 h in the FBS-free DMEM4500 media, breast cancer cells were then subjected to respective incremental solid stress pressures for a duration of 16 h. Subsequently, MDA-MB-231 cells were harvested for the following experiments: cell viability assays, Western blot, RNA Sequencing, RT-qPCR, cell migration assay and invasion assay.

### Cell culture

MDA-MB-231 is a triple negative breast cancer cell line obtained from the American Type Culture Collection (ATCC, HTB-26). The cells were cultured and maintained in Gibco Dulbecco’s Modified Eagle Medium (DMEM) with L-glutamine and high glucose (4.5 g/L Glucose) (Sigma Life Sciences, D5648) supplemented with 10% Fetal Bovine Serum (FBS) (Gibco, A5256701) and Normocin (InvivoGen, ant-nr-05) at 37°C, 5% CO_2_.

### 2D *In vitro* compression device

The 2D *in vitro* compression setup utilises a flat-bottom cup that houses adjustable metal coins to apply a uniform, constant and predefined pressure on monolayer MDA-MB-231 cells that were seeded in a 6-well Transwell 24 mm insert with a 0.4 µm pore diameter Polyester Membrane (Corning Incorporated, 3450). See Figure 1A.

### Application of solid stress compression

MDA-MB-231 cells were seeded (4 × 10^5^ cells) into the upper chambers of 6-well Transwell Polyester Membrane (PET) inserts. The lower chambers of the Transwell are filled with 2.5 mL of DMEM 10% FBS. Right after seeding, the Transwell plate is gently shaken to ensure cells are dispersed equally across the Transwell. Seeded cells are then left to adhere and achieve near confluency where after which, the media is changed to DMEM4500 without FBS. Prior to compression, 2% agarose (1st Base, BIO-1000) disks of roughly 3 mm in thickness and 24 mm in diameter are gently laid upon monolayer cells across all experimental conditions from uncompressed 0.0 Pa (control) to compressed 386.5 Pa, 773.0 Pa and 1546.0 Pa. After which, weighted pistons corresponding to the respective pressures of 386.5 Pa, 773.0 Pa and 1546.0 Pa are then gently placed on the agarose disk and incubated for 16 h at 37°C, 5% CO_2_.

### Weight of compression device components

The setup for cell compression is described in the legend of Figure 1A. The weight needed in grams to exert the respective compressive pressures over a transwell of 24 mm in diameter are 17.48 g, 34.97 g, 69.64 g for 386.5 Pa, 773.0 Pa and 1646.0 Pa, respectively. The formula used to calculate the weights needed to achieve the respective pressures are (a) Pressure (Pa) = Force (N)/area (m^2^); (b) Force (N) = [mass (kg) x Acc (10 m/s^2^)]. E.g., 773 Pa = [mass (kg) x 10 m/s^2^]/π x 0.012^2^).

### Western blot

Cells were harvested and lysed in Lysis Buffer containing 25 mM Hepes (Sigma, H-4034) pH 7.5, 0.3 M sodium chloride (Merck, 7647-14-5), 1 mM magnesium chloride (Merck, 7786-30-3), 1 mM EGTA (Sigma, 324626), 20 mM β-glycerol phosphate (Sigma, 50020), 1 mM sodium vandate (Sigma, 13721-39-6), 10 mM sodium fluoride (Sigma, S1504), 5% glycerol (Affymetrix, 56-81-5), 0.5% Triton-X (Bio-Rad, 1610407), containing phosphatase inhibitors, PhosSTOP (Roche, PHOSS-RO), and Protease Inhibitor (Roche, 11836170001). 50 μg of whole cell lysates were loaded to each well for SDS-PAGE for separation of proteins and followed by Western transfer and blotting. See below for antibodies used.

**Table:** 

Primary antibodies	Company	Species	Dilution
Phosphorylated Akt Serine-473 (D9E XP 4060)	Cell Signaling	Rabbit	1:1000 in 5% BSA
Phosphorylated Akt Threonine-308 (244F9)	Cell Signaling	Rabbit	1:1000 in 5% BSA
Total Akt (9272)	Cell Signaling	Rabbit	1:1000 in 5% Skimmed milk
Phosphorylated GSK-3β Serine-9 (9336S)	Cell Signaling	Rabbit	1:1000 in 5% BSA
Total GSK-3β (9332)	Cell Signaling	Rabbit	1:1000 in 5% Skimmed milk
Phosphorylated STAT3 Tyrosine-705 (D3A7) XP 9145	Cell Signaling	Rabbit	1:1000 in 5% BSA
Total STAT3 (124H6) 9139	Cell Signaling	Mouse	1:1000 in 5% Skimmed Milk
Interleukin-6 (D3K2N)	Cell Signaling	Rabbit	1:1000 in 5% Skimmed Milk
SNAI1 (C15D3)	Cell Signaling	Rabbit	1:1000 in 5% Skimmed Milk
GAPDH (2118)	Cell Signaling	Rabbit	1:4000 in 5% Skimmed milk

Secondary Antibodies	Company	Species	Dilution
Anti-Rabbit IgG, HRP Conjugated (P0448)	Dako Cytomation	Goat	1:4000 in 5% skimmed milk
Anti-Mouse IgG, HRP Conjugated (P0447)	Dako Cytomation	Goat	1:4000 in 5% skimmed milk

### Total RNA preparation for RNA sequencing

A total of six independent solid stress compression experiments (n = 6) at four different compression pressures (0.0 Pa, 386.5 Pa, 773.0 Pa, 1546.0 Pa) were conducted using MDA-MB-231 cells. Total RNA was extracted using RNeasy Plus Mini Kit (Qiagen, 74134). Total RNA samples were then checked for the estimated RNA purity (A260/280) and concentration using the NanoDrop 2000 (ThermoFisher) before being stored at −80°C. RNA Sequencing was performed by Novogene (https://www.novogene.com/us-en/).

### RNA Sequencing Analysis


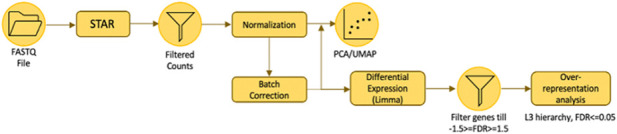


The workflow of RNA Sequencing Analysis is illustrated above. The analysis was conducted by our collaborators (Corinna Goh and Keng-Hwee Chiam) from A*STAR Bioinformatics Institute. The quality of the raw RNA-seq data was evaluated with FastQC (http://www.bioinformatics.babraham.ac.uk/projects/fastqc/). Genome indexes were generated using STAR version 2.7.9a with the comprehensive Gencode v39 gene annotation (Dobin et al., 2013). Raw paired-end FASTQ reads were aligned to the human reference genome GRCh38 and then quantified using STAR aligner (Dobin et al., 2013). They carried out low-counts filtering, count normalisation using the trimmed mean of M-values (TMM) method and Log_2_-transformed the counts per million (CPM) values in *edgeR* version 3.40.2 (Robinson et al., 2020). Since UMAP projections of the principal components (PCA-UMAP) indicated mild batch effects, batch correction was performed using *removeBatchEffect* function in *limma* version 3.54.2 on the Log-CPM values, where the batch variable was specified as the biological replicate (Ritchie et al., 2015).

Differential expression analysis was done using *limma* with *voom* transformation to obtain Log_2_ fold changes (LFCs) for 1546.0 Pa *versus* 0.0 Pa, 773.0 Pa *versus* 0.0 Pa and 386.5 Pa *versus* 0.0 Pa (Law et al., 2014). Over-representation analysis (ORA) was done with pre-defined gene sets in *gprofiler2* version 0.2.1, on a subset of differential genes which fulfilled the criteria of having an absolute LFC higher than or equal to 1.5, and an adjusted p-value of<=0.05 (Kolberg et al., 2020). Gene sets with Benjamini–Hochberg false discovery rate (FDR) below 0.05 were deemed as significantly enriched. To determine the direction of regulation of these enrichments, LFCs were sorted into ranked gene lists for gene set enrichment analysis (GSEA) implemented in *clusterProfiler* version 4.6.2, using only the significant gene sets (Wu et al., 2021). A positive normalised enrichment score (NES) would indicate upregulation of the gene set and *vice versa* for a negative NES.

### Reverse transcription-quantitative polymerase chain reaction

Quantitative PCR was performed using the Taq™ Universal SYBR® Green Supermix (Bio-Rad, 1725121) on the Bio-Rad CFX96 Touch Real-Time PCR Detection System. Thermal cycling conditions are summarised in below. Primer sequences and PrimerBank ID used are summarised in the below. All primers have been previously validated by other studies. GAPDH is the housekeeping gene used to normalise the data.

## Thermal cycling protocol

**Table:** 

Thermal cycling protocol CFX96 touch			
Denaturation cDNA	95°C	30 seconds	
Denaturation cDNA	95°C	5 Seconds	x 40 Cycles
Annealing/Extension	60°C	30 Seconds	
Melt Curve	65–95°C	0.5°C increment/5 s	

See below for the list of primers used to validate RNA sequencing targets and the list of genes with their respective primer direction, PrimerBankID and sequence in 5’->3′ direction. GAPDH was used to normalise the data.

**Table:** 

Gene Name	Primer direction	Primer Bank ID	Sequence (5′ → 3′)
IL6	Forward	224831235c1	ACTCACCTCTTCAGAACG AATTG
IL6	Reverse	224831235c1	CCATCTTTGGAAGGTTCA GGTTG
IL6R	Forward	332309221c1	CCCCTCAGCAATGTTGTT TGT
IL6R	Reverse	332309221c1	CTCCGGGACTCGTAACTGG
SNAI1	Forward	301336132c1	TCGGAAGCCTAACTACAG CGA
SNAI1	Reverse	301336132c1	AGATGAGCATTGGCAGC GAG
MYC	Forward	239582723c1	GGCTCCTGGCAAAAGGTCA
MYC	Reverse	239582723c1	CTGCGTAGTTGTGCTGAT GT
COL1A1	Forward	110349771c2	GTGCGATGACGTGATCTG TGA
COL1A1	Reverse	110349771c2	CGGTGGTGTTTTGTGGT TGG
LOX	Forward	296010939c1	CGGCGGAGGAAAACATG TCT
LOX	Reverse	296010939c1	TCGGCTGGGTAAGAAATC TGA
ACTBL2	Forward	144922730c2	CTCGACACCAGGGCGTT ATG
ACTBL2	Reverse	144922730c2	CCACTCCATGCTCGATAG GAT
GAPDH	Forward	378404907c3	CTGGGCTACACTGAGCACC
GAPDH	Reverse	378404907c3	AAGTGGTCGTTGAGGGCA ATG

### Scratch-wound assay

MDA-MB-231 cells were seeded in transwells and grew till confluent. A ‘scratch’ wound was created, using a thin loading tip, across all the transwells. Images were captured using Live Cell Observer II (Zeiss) for the 0-h time point. Five different images were taken along the scratch wound for each experimental condition. The cells were then subjected to compression. After 16 h, the respective weights were removed, and the wound was imaged again. Similarly, five images were taken along the scratch wound. The wound area closure for each of the five images were calculated using ImageJ.

### Invasion assay

Matrigel was coated onto an 8.0 µm transwell plate (Corning Incorporated, 3428). MDA-MB-231 cells were seeded at the density of 5 × 10^5^ cells/transwell in serum free DMEM4500 media. Solid stress compression was applied as in the migration assays. Cells which invaded through the Matrigel were quantified using crystal violet.

### Statistical analysis

Statistical analyses were conducted using a two-tailed student T-test. Error bars represent standard deviation (SD). (*) p ≤ 0.05 and (**) p ≤ 0.01 and (N.S) Not Significant.

## Data Availability

The datasets presented in this study can be found in online repositories. The names of the repository/repositories and accession number(s) can be found below: https://www.ncbi.nlm.nih.gov/geo/,GSE282791.
