# Supplementary material for: Solid stress compression enhances breast cancer cell migration through the upregulation of Interleukin-6
Source: Front Cell Dev Biol. 2025 Apr 30;13:1541953. doi: 10.3389/fcell.2025.1541953 (PMC12077316; doi:10.3389/fcell.2025.1541953)
Supplement: Supplementary file 1 [file DataSheet1.pdf]

Supplementary Material

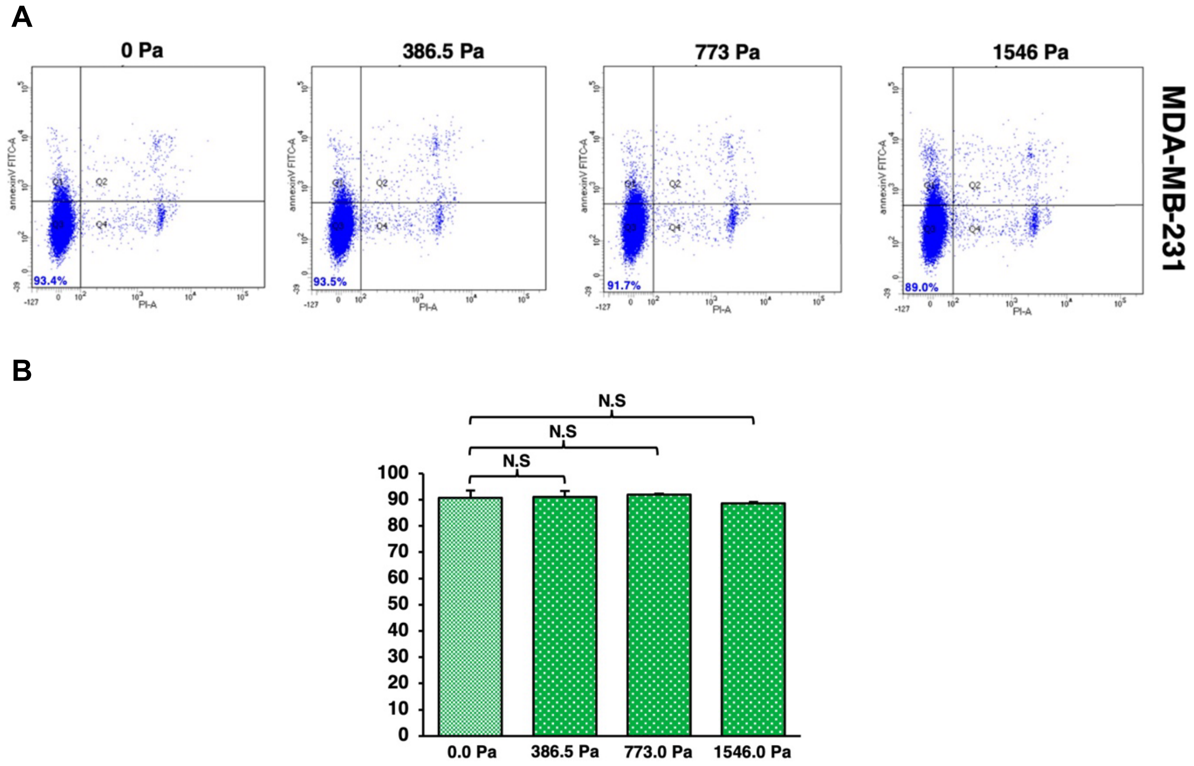

Supplementary Figure 1. Cell Viability Assay of MDA-MB-231 breast cancer cells under incremental solid stress compression for 16 hours. **(A)** Flow cytometric analysis of MDA-MB-231 cells using Annexin V-FITC and Propidium Iodide staining. Representative dot-plot highlighting viable cells (Bottom left quadrant), early apoptotic (Top left quadrant) and late apoptotic (Top right quadrant) MDA-MB-231 cells. The mean percentage of viable cells across three independent experiments (n=3) is labelled in the lower left quadrant which represents viable cells (Annexin V-FITC and PI negative). **(B)** Mean percentage of cell viability from respective conditions from three independent experiments (n=3). Error bars represent standard deviation. Statistical analysis was done using student T-test 2-tailed. (*) $p \leq 0.05$ and (**) $p \leq 0.01$ and (N.S) Not Significant

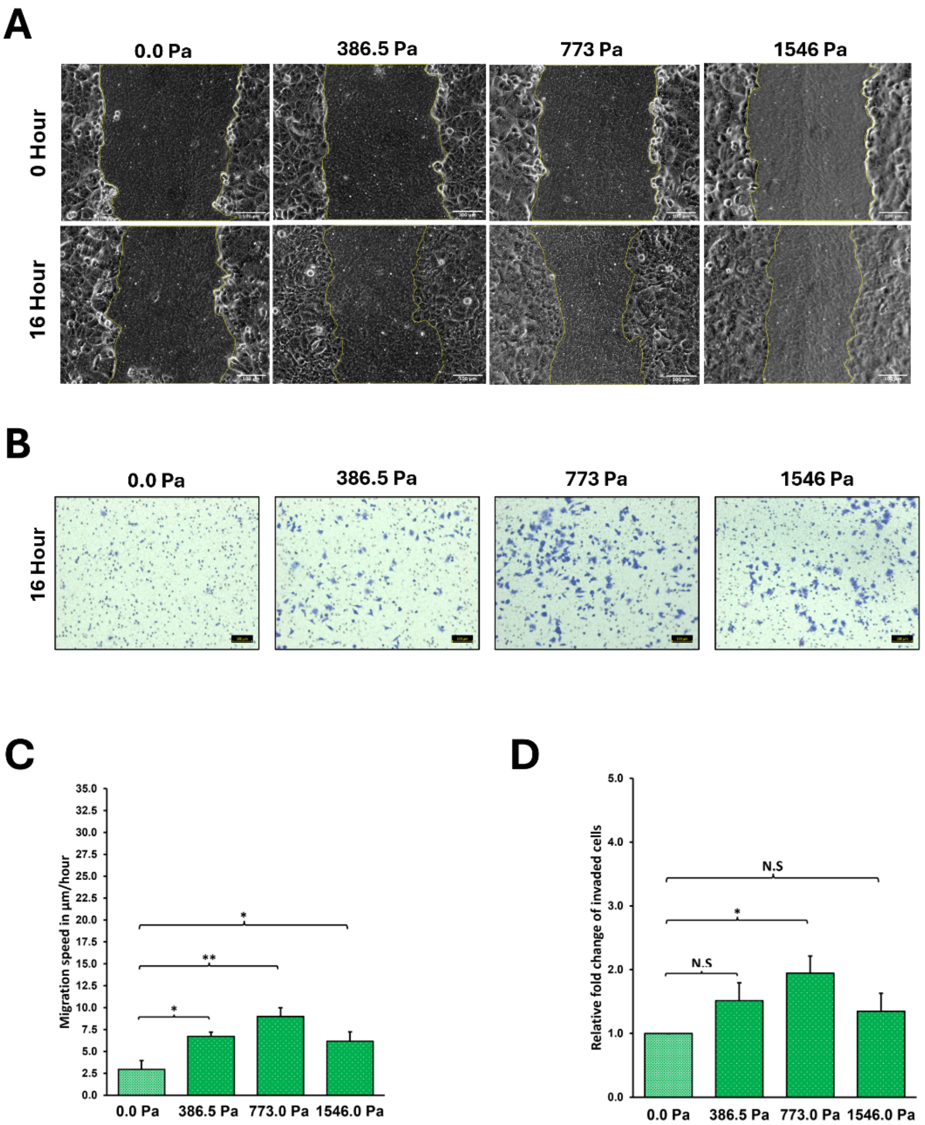

Supplementary Figure 2. Migration and Invasion Assay of MCF-7 non-invasive breast cancer cells under incremental solid stress compression **(A)** Representative Scratch-Wound Assay images highlighting the changes in MCF-7 cell migration under 16 hours compression at various solid stress levels – 386.5 Pa, 773.0 Pa and 1546.0 Pa. Uncompressed cells, 0.0 Pa, were covered with an agarose disk only. Scale bar: 100 µm. **(B)** Representative Invasion Assay images under 16 hours compression at various solid stress levels – 386.5 Pa, 773.0 Pa and 1546.0 Pa. Uncompressed cells, 0.0 Pa, were covered with an agarose disk only. Scale bar: 100 µm. **(C)** Analysed by Image Lab, bar graph represents mean migration speed in µm/hour from three independent experiments (n=3). Error bars represent standard deviation. Statistical analysis was done using 2-tailed student T-test. (*) $p \leq 0.05$, (**) $p \leq 0.01$ and (N.S) Not Significant. **(D)** Fold changes of number of invaded cells under compression as compared to uncompressed cells. Invaded cells were stained with 0.1% crystal violet solution and absorbance at 590nm was recorded. Bar graph represents the mean number of invaded cells obtained from three independent experiments (n=3). Error bars represent standard deviation. Statistical analysis was done using 2-tailed student T-test. (*) $p \leq 0.05$, (**) $p \leq 0.01$ and (N.S) Not Significant.

MDA-MB-231
386.5 Pa Vs 0.0 Pa

A

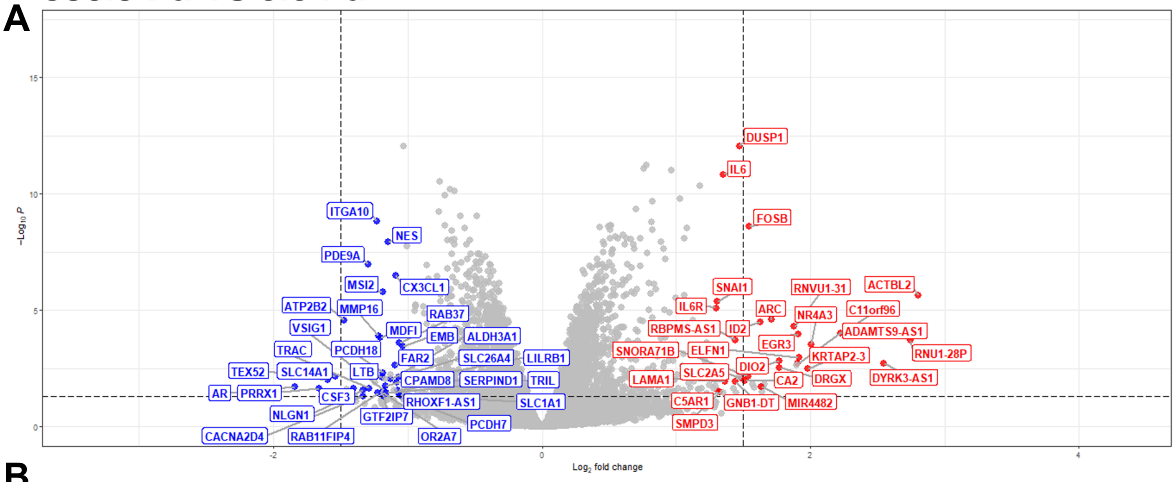

B

Top 40 Upregulated Genes 386.5 Pa Vs 0.0 Pa

Rank	Gene Ensembl	Gene Name	Log2 FC	adj. P.Val
1	ENSG00000169067	ACTBL2	2.80	0.0000022
2	ENSG00000206588	RNU1-28P	2.75	0.00019758
3	ENSG00000237605	DYRK3-AS1	2.54	0.00186808
4	ENSG00000187479	C11orf96	2.22	0.00010063
5	ENSG00000119508	NR4A3	2.01	0.00029187
6	ENSG00000212724	KRTAP2-3	1.98	0.00330878
7	ENSG00000225968	ELFN1	1.92	0.00104684
8	ENSG00000179388	EGR3	1.91	0.00010708
9	ENSG00000241158	ADAMTS9-AS1	1.90	0.00196482
10	ENSG00000262202	Novel Transcript	1.90	0.00223212
11	ENSG00000270722	RNVU1-31	1.88	0.0000491
12	ENSG00000165606	DRGX	1.77	0.00286395
13	ENSG00000211448	DIO2	1.77	0.00147365
14	ENSG00000198576	ARC	1.71	0.0000254
15	ENSG00000205959	Novel Transcript	1.66	0.01503286
16	ENSG00000229808	Novel Transcript	1.64	0.019164
17	ENSG00000229021	Novel Transcript	1.64	0.0308149
18	ENSG00000266852	MIR4482	1.63	0.01872889
19	ENSG00000115738	ID2	1.63	0.0000322
20	ENSG00000289602	Novel Transcript	1.58	2.12E-02
21	ENSG00000288919	Novel Transcript	1.57	0.0152681
22	ENSG00000125740	FOSB	1.54	2.41E-09
23	ENSG00000235408	SNORA71B	1.54	0.00668709
24	ENSG00000104267	CA2	1.51	0.01033447
25	ENSG00000142583	SLC2A5	1.49	0.0080897
26	ENSG00000262147	Novel Transcript	1.48	0.00469891
27	ENSG00000120129	DUSP1	1.47	8.8E-13
28	ENSG00000272277	Novel Transcript	1.47	0.00717079
29	ENSG00000223884	Novel Transcript	1.45	0.0345009
30	ENSG00000231050	GNB1-DT	1.44	0.01189606
31	ENSG00000254109	RBPMS-AS1	1.44	0.00018412
32	ENSG00000286416	Novel Transcript	1.43	0.02284117
33	ENSG00000101680	LAMA1	1.37	0.01133761
34	ENSG00000289915	Novel Transcript	1.35	0.03510335
35	ENSG00000136244	IL6	1.35	1.46E-11
36	ENSG00000103056	SMPD3	1.32	0.03248172
37	ENSG00000197405	C5AR1	1.31	0.02966009
38	ENSG00000124216	SNAI1	1.30	0.00000416
39	ENSG00000160712	IL6R	1.30	0.00000783
40	ENSG00000289393	Novel Transcript	1.29	0.0182001

Top 40 Downregulated Genes 386.5 Pa Vs 0.0 Pa

Rank	Gene Ensembl	Gene Name	Log2 FC	adj. P.Val
1	ENSG00000169083	AR	-1.84	0.01915296
2	ENSG00000116132	PRRX1	-1.66	0.02290009
3	ENSG00000283297	TEX52	-1.60	0.00943354
4	ENSG00000141469	SLC14A1	-1.54	0.00685286
5	ENSG00000157087	ATP2B2	-1.47	0.0000268
6	ENSG00000241269	Novel Transcript	-1.46	0.02548551
7	ENSG00000108342	CSF3	-1.40	0.02267165
8	ENSG00000151062	CACNA2D4	-1.33	0.04884672
9	ENSG00000169760	NLGN1	-1.33	0.02637177
10	ENSG00000160191	PDE9A	-1.29	1.03E-07
11	ENSG00000277734	TRAC	-1.29	0.02181317
12	ENSG00000272129	Novel Transcript	-1.24	0.01295993
13	ENSG00000143127	ITGA10	-1.23	1.42E-09
14	ENSG00000131242	RAB11FIP4	-1.22	0.03574757
15	ENSG00000156103	MMP16	-1.22	0.00011993
16	ENSG00000112559	MDFI	-1.21	0.00015159
17	ENSG00000227507	LTB	-1.19	0.00750404
18	ENSG00000243896	OR2A7	-1.19	0.03291528
19	ENSG00000153944	MSI2	-1.19	0.00000163
20	ENSG00000189184	PCDH18	-1.18	0.00480734
21	ENSG00000254363	Novel Transcript	-1.18	0.03330459
22	ENSG00000227038	GTF2IP7	-1.18	0.04723891
23	ENSG00000101842	VSIG1	-1.18	0.02612505
24	ENSG00000160111	CPAMD8	-1.17	0.01767556
25	ENSG00000237094	Novel Transcript	-1.17	0.00859702
26	ENSG00000255690	TRIL	-1.16	0.03465753
27	ENSG00000132688	NES	-1.15	1.18E-08
28	ENSG00000230499	Novel Transcript	-1.13	0.00569731
29	ENSG00000108602	ALDH3A1	-1.13	0.00893025
30	ENSG00000230834	Novel Transcript	-1.10	0.02643432
31	ENSG00000064763	FAR2	-1.10	0.0022675
32	ENSG00000006210	CX3CL1	-1.09	3.16E-07
33	ENSG00000104972	LILRB1	-1.09	0.0127126
34	ENSG00000099937	SERPIND1	-1.07	0.0278554
35	ENSG00000091137	SLC26A4	-1.07	0.00719192
36	ENSG00000106688	SLC1A1	-1.06	0.04146435
37	ENSG00000172794	RAB37	-1.06	0.0002477
38	ENSG00000258545	RHOXF1-AS1	-1.05	0.04366709
39	ENSG00000169851	PCDH7	-1.04	0.04993635
40	ENSG00000170571	EMB	-1.04	0.00034925

Supplementary Figure 3. Significant transcriptomic alterations in MDA-MB-231 cells under 386.5 Pa. **(A)** Volcano plot illustrating the top 40 highly upregulated genes (red) and highly downregulated genes (blue) in MDA-MB-231 cells under 386.5 Pa compared with uncompressed condition. **(B)** Table listing the highly upregulated and downregulated genes and their respective Log_2 fold changes and adjusted p-values.

MDA-MB-231
773.0 Pa Vs 0.0 Pa

A

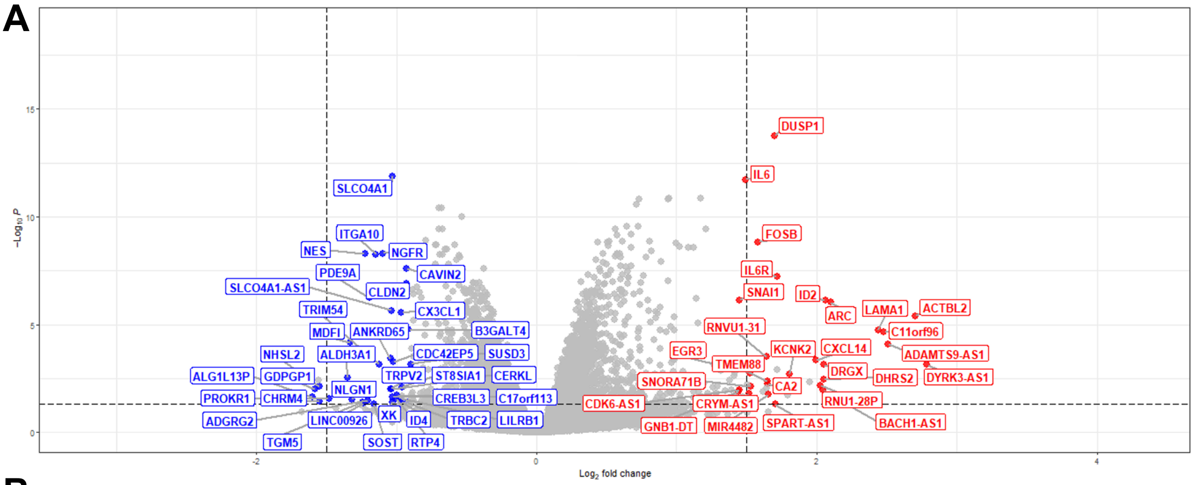

B

Top 40 Upregulated Genes 773.0 Pa Vs 0.0 Pa

Rank	Gene Ensembl	Gene Name	Log2 FC	adj. P.Val
1	ENSG00000237605	DYRK3-AS1	2.79	0.00072956
2	ENSG00000169067	ACTBL2	2.70	0.00000399
3	ENSG00000241158	ADAMTS9-AS1	2.51	0.0000819
4	ENSG00000187479	C11orf96	2.48	0.0000229
5	ENSG00000101680	LAMA1	2.44	0.000018
6	ENSG00000198576	ARC	2.10	9.38E-07
7	ENSG00000115738	ID2	2.06	7.47E-07
8	ENSG00000165606	DRGX	2.05	0.00072956
9	ENSG00000100867	DHRS2	2.05	0.00349949
10	ENSG00000232118	BACH1-AS1	2.05	0.01080341
11	ENSG00000206588	RNU1-28P	2.03	0.00640553
12	ENSG00000145824	CXCL14	1.99	0.00044085
13	ENSG00000289602	Novel Transcript	1.95	0.00439109
14	ENSG00000288919	Novel Transcript	1.83	0.00446268
15	ENSG00000082482	KCNK2	1.81	0.0019004
16	ENSG00000229021	Novel Transcript	1.79	0.01881446
17	ENSG00000262147	Novel Transcript	1.72	0.00124468
18	ENSG00000160712	IL6R	1.72	6.18E-08
19	ENSG00000120664	SPART-AS1	1.70	0.0492877
20	ENSG00000120129	DUSP1	1.70	1.76E-14
21	ENSG00000266852	MIR4482	1.66	0.01727815
22	ENSG00000167874	TMEM88	1.65	0.0042317
23	ENSG00000104267	CA2	1.65	0.00551536
24	ENSG00000262202	Novel Transcript	1.64	0.00854278
25	ENSG00000270722	RNVU1-31	1.64	0.00031546
26	ENSG00000223461	Novel Transcript	1.62	0.01327743
27	ENSG00000125740	FOSB	1.58	1.48E-09
28	ENSG00000235408	SNORA71B	1.53	0.0073327
29	ENSG00000179388	EGR3	1.53	0.00174571
30	ENSG00000189149	CRYM-AS1	1.52	0.01617668
31	ENSG00000272277	Novel Transcript	1.51	0.0061933
32	ENSG00000205959	Novel Transcript	1.49	0.03072528
33	ENSG00000136244	IL6	1.49	1.94E-12
34	ENSG00000229808	Novel Transcript	1.48	0.03684889
35	ENSG00000267898	Novel Transcript	1.48	0.02312795
36	ENSG00000237819	CDK6-AS1	1.45	0.01043547
37	ENSG00000124216	SNAI1	1.45	7.18E-07
38	ENSG00000231050	GNB1-DT	1.44	0.01243397
39	ENSG00000286416	Novel Transcript	1.43	0.02338724
40	ENSG00000277383	Novel Transcript	1.42	0.00011594

Top 40 Downregulated Genes 773.0 Pa Vs 0.0 Pa

Rank	Gene Ensembl	Gene Name	Log2 FC	adj. P.Val
1	ENSG00000253981	ALG1L13P	-1.60	0.02339504
2	ENSG00000183208	GDPGP1	-1.58	0.00990629
3	ENSG00000230834	Novel Transcript	-1.55	0.00379238
4	ENSG00000204131	NHSL2	-1.55	0.00740511
5	ENSG00000169618	PROKR1	-1.55	0.03552198
6	ENSG00000180720	CHRM4	-1.48	0.0265135
7	ENSG00000108602	ALDH3A1	-1.35	0.00280692
8	ENSG00000112559	MDFI	-1.33	0.0000697
9	ENSG00000169760	NLGN1	-1.32	0.02881908
10	ENSG00000104055	TGM5	-1.24	0.03986557
11	ENSG00000247982	LINC00926	-1.23	0.04545728
12	ENSG00000132688	NES	-1.22	5.24E-09
13	ENSG00000173698	ADGRG2	-1.20	0.03201911
14	ENSG00000160191	PDE9A	-1.19	5.79E-07
15	ENSG00000187185	Novel Transcript	-1.18	0.00683285
16	ENSG00000167941	SOST	-1.16	0.04981004
17	ENSG00000143127	ITGA10	-1.15	5.6E-09
18	ENSG00000138100	TRIM54	-1.12	0.00069634
19	ENSG00000064300	NGFR	-1.10	5E-09
20	ENSG00000235098	ANKRD65	-1.04	0.00037963
21	ENSG00000187688	TRPV2	-1.04	0.00955515
22	ENSG00000188452	CERKL	-1.03	0.00976786
23	ENSG00000232803	SLCO4A1-AS1	-1.03	0.00000227
24	ENSG00000060566	CREB3L3	-1.03	0.0230924
25	ENSG00000047597	XK	-1.03	0.03795357
26	ENSG00000101187	SLCO4A1	-1.03	1.29E-12
27	ENSG00000167617	CDC42EP5	-1.02	0.00052927
28	ENSG00000136514	RTP4	-1.00	0.02835802
29	ENSG00000267221	C17orf113	-0.99	0.01951197
30	ENSG00000104972	LILRB1	-0.97	0.02663423
31	ENSG00000172201	ID4	-0.97	0.03873304
32	ENSG00000006210	CX3CL1	-0.96	0.0000028
33	ENSG00000230499	Novel Transcript	-0.96	0.02069431
34	ENSG00000111728	ST8SIA1	-0.96	0.00773233
35	ENSG00000168497	CAVIN2	-0.93	2.46E-08
36	ENSG00000165376	CLDN2	-0.93	1.23E-07
37	ENSG00000211772	TRBC2	-0.92	0.0402661
38	ENSG00000261663	Novel Transcript	-0.92	0.04454221
39	ENSG00000235863	B3GALT4	-0.91	0.000016
40	ENSG00000157303	SUSD3	-0.90	0.00068129

Supplementary Figure 4. Significant transcriptomic alterations in MDA-MB-231 cells under 773.0 Pa. **(A)** Volcano plot illustrating the top 40 highly upregulated genes (red) and highly downregulated genes (blue) in MDA-MB-231 cells under 773.0 Pa compared with uncompressed condition. **(B)** Table listing the highly upregulated and downregulated genes and their respective Log$_2$ fold changes and adjusted p-values.

MDA-MB-231
1546.0 Pa Vs 0.0 Pa

A

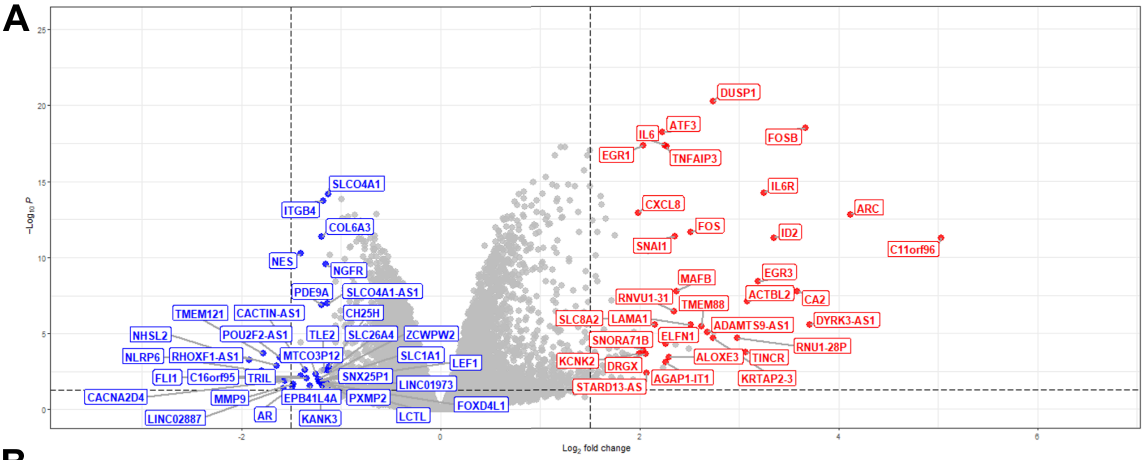

B

Top 40 Upregulated Genes 1546.0 Pa Vs 0.0 Pa

Rank	Gene Ensembl	Gene Name	Log2 FC	adj. P.Val
1	ENSG00000187479	C11orf96	5.03	5.33E-12
2	ENSG00000198576	ARC	4.12	1.57E-13
3	ENSG00000237605	DYRK3-AS1	3.71	0.00000286
4	ENSG00000125740	FOSB	3.67	3.25E-19
5	ENSG00000104267	CA2	3.59	1.73E-08
6	ENSG00000115738	ID2	3.35	5.49E-12
7	ENSG00000160712	IL6R	3.25	5.74E-15
8	ENSG00000179388	EGR3	3.19	4.15E-09
9	ENSG00000169067	ACTBL2	3.08	8.61E-08
10	ENSG00000223573	TINCR	3.07	0.00017125
11	ENSG00000206588	RNU1-28P	2.98	0.0000215
12	ENSG00000288919	Novel Transcript	2.75	0.00000857
13	ENSG00000120129	DUSP1	2.74	5.88E-21
14	ENSG00000212724	KRTAP2-3	2.74	0.0000209
15	ENSG00000241158	ADAMTS9-AS1	2.68	0.00000812
16	ENSG00000167874	TMEM88	2.62	0.00000336
17	ENSG00000170345	FOS	2.51	2.38E-12
18	ENSG00000101680	LAMA1	2.51	0.00000288
19	ENSG00000289602	Novel Transcript	2.50	0.00010384
20	ENSG00000204103	MAFB	2.37	1.71E-08
21	ENSG00000262202	Novel Transcript	2.36	0.0000556
22	ENSG00000124216	SNAI1	2.36	4.29E-12
23	ENSG00000270722	RNVU1-31	2.35	3.57E-07
24	ENSG00000179148	ALOXE3	2.29	0.00036777
25	ENSG00000118503	TNFAIP3	2.27	5.42E-18
26	ENSG00000235529	AGAP1-IT1	2.26	0.0008541
27	ENSG00000225968	ELFN1	2.26	0.0000481
28	ENSG00000136244	IL6	2.25	4.4E-18
29	ENSG00000289987	Novel Transcript	2.24	0.0018606
30	ENSG00000162772	ATF3	2.23	5.86E-19
31	ENSG00000118160	SLC8A2	2.16	0.00000277
32	ENSG00000274213	Novel Transcript	2.09	3.97E-11
33	ENSG00000236581	STARD13-AS	2.07	0.00410656
34	ENSG00000165606	DRGX	2.06	0.00022722
35	ENSG00000120738	EGR1	2.04	4.4E-18
36	ENSG00000235408	SNORA71B	2.03	0.00013692
37	ENSG00000082482	KCNK2	1.99	0.00023114
38	ENSG00000169429	CXCL8	1.99	1.22E-13
39	ENSG00000236453	Novel Transcript	1.95	0.00000832
40	ENSG00000234424	Novel Transcript	1.94	0.00055643

Top 40 Downregulated Genes 1546.0 Pa Vs 0.0 Pa

Rank	Gene Ensembl	Gene Name	Log2 FC	adj. P.Val
1	ENSG00000204131	NHSL2	-1.92	0.00052221
2	ENSG00000174885	NLRP6	-1.80	0.00276655
3	ENSG00000184986	TMEM121	-1.78	0.00021107
4	ENSG00000258545	RHOXF1-AS1	-1.65	0.00129838
5	ENSG00000259265	Novel Transcript	-1.62	0.01374716
6	ENSG00000151702	FLI1	-1.62	0.00990372
7	ENSG00000254887	POU2F2-AS1	-1.61	0.00038184
8	ENSG00000280279	LINC02887	-1.58	0.04218561
9	ENSG00000151062	CACNA2D4	-1.56	0.01292447
10	ENSG00000241269	Novel Transcript	-1.52	0.01016071
11	ENSG00000169083	AR	-1.49	0.03146706
12	ENSG00000100985	MMP9	-1.48	0.02165558
13	ENSG00000260456	C16orf95	-1.41	0.00642573
14	ENSG00000132688	NES	-1.40	5.46E-11
15	ENSG00000256663	Novel Transcript	-1.40	0.0000401
16	ENSG00000226800	CACTIN-AS1	-1.36	0.00267606
17	ENSG00000255690	TRIL	-1.34	0.00862028
18	ENSG00000129595	EPB41L4A	-1.31	0.02725359
19	ENSG00000198744	MTCO3P12	-1.25	0.00489316
20	ENSG00000206559	ZCWPW2	-1.23	0.00972875
21	ENSG00000138795	LEF1	-1.22	0.01392585
22	ENSG00000236809	SNX25P1	-1.22	0.01426762
23	ENSG00000106688	SLC1A1	-1.20	0.01227224
24	ENSG00000270179	Novel Transcript	-1.20	0.00279467
25	ENSG00000184492	FOXD4L1	-1.19	0.01775295
26	ENSG00000163359	COL6A3	-1.19	4.59E-12
27	ENSG00000160191	PDE9A	-1.19	1.35E-07
28	ENSG00000186994	KANK3	-1.18	0.02628943
29	ENSG00000132470	ITGB4	-1.18	1.86E-14
30	ENSG00000065717	TLE2	-1.17	0.00241385
31	ENSG00000188501	LCTL	-1.15	0.02486347
32	ENSG00000064300	NGFR	-1.15	2.95E-10
33	ENSG00000204283	LINC01973	-1.14	0.01747588
34	ENSG00000266601	Novel Transcript	-1.14	0.04980675
35	ENSG00000235959	Novel Transcript	-1.14	0.02403913
36	ENSG00000232803	SLCO4A1-AS1	-1.13	1.06E-07
37	ENSG00000091137	SLC26A4	-1.13	0.00290923
38	ENSG00000101187	SLCO4A1	-1.13	7.07E-15
39	ENSG00000138135	CH25H	-1.12	0.0015627
40	ENSG00000176894	PXMP2	-1.11	0.02508702

Supplementary Figure 5. Significant transcriptomic alterations in MDA-MB-231 cells under 1546.0 Pa. **(A)** Volcano plot illustrating the top 40 highly upregulated genes (red) and highly downregulated genes (blue) in MDA-MB-231 cells under 1546.0 Pa compared with uncompressed condition. **(B)** Table listing the highly upregulated and downregulated genes and their respective Log_2 fold changes and adjusted p-values.

A

	386.5 Pa Vs 0.0 Pa	773.0 Pa v 0.0 Pa	1546.0 Pa v 0.0 Pa
COL1A1	0.644	0.610	0.502
LOX	0.348	0.419	0.445
ACTBL2	2.803	2.703	3.082

B

	386.5 Pa Vs 0.0 Pa	773.0 Pa v 0.0 Pa	1546.0 Pa v 0.0 Pa
CXCL8	0.329	0.487	1.988

C

	386.5 Pa Vs 0.0 Pa	773.0 Pa v 0.0 Pa	1546.0 Pa v 0.0 Pa
PIEZO1	-0.271	-0.177	-0.096
KCNK2	0.809	1.808	1.993

Log_2 Fold Change

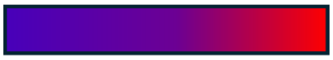

-1.0 1.0

White Log_2 Fold Changes are significant (P<=0.05)

Yellow underlined Log_2 Fold Changes are not significant (P>0.05)

Supplementary Figure 6. Heatmap and Log_2 Fold Changes of other targets mentioned in the study. **(A)** Log_2 Fold changes of targets validated by RT-qPCR but not pathway-specific: COL1A1 (Collagen Type I Alpha 1 Chain), LOX (Lysyl Oxidase), ACTBL2 (Actin Beta Like 2). **(B)** Log_2 Fold change of CXCL8 (CXC motif chemokine ligand 8). **(C)** Log_2 Fold changes of PIEZO1 (Piezo Type Mechanosensitive Ion Channel Component 1) and KCNK2 (Potassium Two Pore Domain Channel Subfamily K Member 2). Log_2 Fold changes in white fonts are significant (p <= 0.05) and Log2 fold changes in yellow underlined fonts are not significant (p > 0.05)
